# Single-cell transcriptomic reveals molecular diversity and developmental heterogeneity of human stem cell-derived oligodendrocyte lineage cells

**DOI:** 10.1038/s41467-021-20892-3

**Published:** 2021-01-28

**Authors:** Xitiz Chamling, Alyssa Kallman, Weixiang Fang, Cynthia A. Berlinicke, Joseph L. Mertz, Prajwal Devkota, Itzy E. Morales Pantoja, Matthew D. Smith, Zhicheng Ji, Calvin Chang, Aniruddha Kaushik, Liben Chen, Katharine A. Whartenby, Peter A. Calabresi, Hai-Quan Mao, Hongkai Ji, Tza-Huei Wang, Donald J. Zack

**Affiliations:** 1 Department of Ophthalmology, Wilmer Eye Institute, Johns Hopkins University School of Medicine, Baltimore, MD 21287 USA; 2 Department of Genetic Medicine, Johns Hopkins University School of Medicine, Baltimore, MD 21287 USA; 3 Department of Biostatistics, Johns Hopkins Bloomberg School of Public Health, Baltimore, MD 21205 USA; 4 Department of Computer Science, University of Miami, Coral Gables, FL 33146 USA; 5 Department of Neurology, Johns Hopkins University School of Medicine, Baltimore, MD 21287 USA; 6 Department of Biomedical Engineering, Johns Hopkins School of Medicine, Baltimore, MD 21205 USA; 7 Department of Mechanical Engineering, Johns Hopkins University, Baltimore, MD 21218 USA; 8 The Solomon H. Snyder Department of Neuroscience, Johns Hopkins University School of Medicine, Baltimore, MD 21205 USA; 9 Translational Tissue Engineering Center, Johns Hopkins School of Medicine, Baltimore, MD 21287 USA; 10 Institute for NanoBioTechnology, Johns Hopkins University, Whiting School of Engineering Baltimore, Maryland, MD 21218 USA; 11 Department of Molecular Biology and Genetics, Johns Hopkins University School of Medicine, Baltimore, MD 21287 USA

**Keywords:** RNA sequencing, Developmental neurogenesis, Astrocyte, Oligodendrocyte, Pluripotent stem cells

## Abstract

Injury and loss of oligodendrocytes can cause demyelinating diseases such as multiple sclerosis. To improve our understanding of human oligodendrocyte development, which could facilitate development of remyelination-based treatment strategies, here we describe time-course single-cell-transcriptomic analysis of developing human stem cell-derived oligodendrocyte-lineage-cells (hOLLCs). The study includes hOLLCs derived from both genome engineered embryonic stem cell (ESC) reporter cells containing an Identification-and-Purification tag driven by the endogenous *PDGFRα* promoter and from unmodified induced pluripotent (iPS) cells. Our analysis uncovers substantial transcriptional heterogeneity of PDGFRα-lineage hOLLCs. We discover sub-populations of human oligodendrocyte progenitor cells (hOPCs) including a potential cytokine-responsive hOPC subset, and identify candidate regulatory genes/networks that define the identity of these sub-populations. Pseudotime trajectory analysis defines developmental pathways of oligodendrocytes vs astrocytes from PDGFRα-expressing hOPCs and predicts differentially expressed genes between the two lineages. In addition, pathway enrichment analysis followed by pharmacological intervention of these pathways confirm that mTOR and cholesterol biosynthesis signaling pathways are involved in maturation of oligodendrocytes from hOPCs.

## Abstract (web summary)

Brain myelinating oligodendrocytes are rare and difficult to isolate, which has limited data on their development. Here the authors develop a reporter for scalable purification of human pluripotent stem cell derived oligodendrocyte lineage cells, and use this to map differentiation using single cell RNA-sequencing,

## Introduction

Myelin, the insulating material that coats and protects axons and enables rapid saltatory conduction, is essential for the health and function of most vertebrate neurons^1^. Myelin disorders, the most common of which is multiple sclerosis, can be inherited or acquired, can occur from diverse etiologies such as genetic mutation, toxic injury, or autoimmune insult, and can often lead to severe disability^2^. Although there are a number of drugs that can modulate the demyelinating process, these drugs are generally not effective at promoting remyelination. Development of remyelination-based therapies, which could have enormous clinical impact, would be greatly aided by an increased understanding of the regulatory pathways and molecular mechanisms involved in the development of oligodendrocytes (OLs), a subtype of glial cells that are responsible for synthesizing and maintaining central nervous system (CNS) myelin. Transcriptomic and regulatory pathway studies, which have led to the discovery of compounds that potentially target myelinogenic oligodendrocytes^3–6^, have predominantly used rodent OPCs and OLs. However, there are fundamental differences between rodent and human OPCs and OLs. For example, over two hundred human OPC genes are not expressed by mouse OPCs^7–9^, and expression of *Ascl1*, which is essential for OL fate induction from mouse neural precursor cells, is dispensable in human cells^8,10^. Therefore, for both improved disease modeling and to support myelination-based drug-discovery efforts, a more detailed transcriptomic analysis using human oligodendrocyte-lineage cells (hOLLCs) would be helpful.

One of the bottlenecks limiting the use of hOLLCs in developmental, transcriptomic, and drug-discovery studies is the challenge of obtaining sufficient numbers of purified cells—primary human OPCs are rare, difficult to isolate, and cannot be expanded following isolation^11^. An alternative to using primary cells is to use human pluripotent stem cell (hPSC)-derived hOPCs, but tracking and isolating large numbers of pure hOPCs from a mixed population of differentiating CNS cells is still technically challenging^12–15^. In this study, we engineered a unique reporter system by knocking-in an identification-and-purification (IAP) reporter sequence at the 3′-end of the endogenous *PDGFRα* locus of a human embryonic stem cell (hESC) line. This reporter system enables scalable differentiation and purification of PDGFRα expressing hOLLCs at various stages of differentiation. The hESC-derived and purified reporter hOLLCs were then used for droplet-based single-cell capture and RNA-sequencing (Drop-seq)^16^ at three different stages of differentiation. A second population of PDGFR*α* expressing cells, derived from an unmodified induced pluripotent stem (iPS) cell line, was also studied. The single-cell RNA-sequencing (scRNA-seq) identified transcriptionally distinct cells within the hOPC populations^16,17^, revealing the genetic diversity of human PDGFR*α*^+^ OPCs and facilitating an in-depth analysis of their differentiation pathways. Analysis of the differentially expressed genes in mature human OLs (hOLs) compared to hOPCs identified pathways that may contribute to hOL maturation. Pharmacological modulation of the implicated pathways validated in human cells a number of regulatory genes and pathways that had been previously identified from murine studies. Also, similar to previous reports, we found that a subset of PDGFR*α*^+^ precursors can give rise to astrocyte-like cells^11,18^, and further identified a subgroup of PDGFRα*-*lineage cells that express mature astrocyte (AS) or oligodendrocyte markers. Taking advantage of the bipotential nature of the reporter cells, we performed pseudotime analysis^19^ to track the differentiation trajectories of the subsets of OLs and astrocytes. This analysis identified genetic factors that are enriched in hOLs or astrocytes, and are potentially involved in regulating human OL vs astrocyte lineage specification.

## Results

### Generation of an OPC differentiation and purification stem cell reporter line

**Fig. 1:**
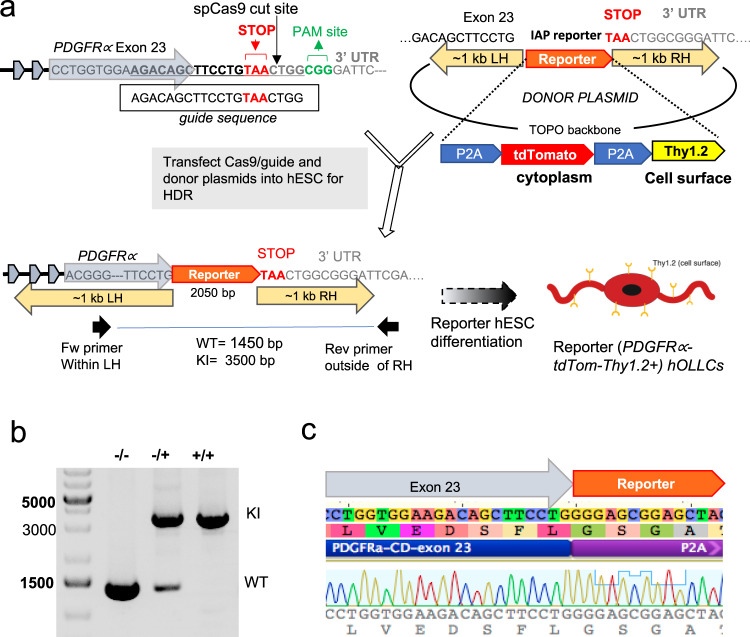
Generation of PDGFRα reporter ES cell line. **a** Schematic diagram of IAP reporter knock-in into the *PDGFRα* locus using CRISPR–Cas9 genome editing. A plasmid containing spCas9 sequence and a guide sequence targeting the stop codon of the *PDGFRα* gene, and a separate “donor” plasmid containing reporter sequence flanked by 1 kb homology arms were transfected into H9 ES cells. Following single-cell passaging, PCR-based genotyping was performed on individual clones using a primer set that spans the reporter sequence (one primer at the homology arm and the other outside the homology arm). **b** A representative PCR gel image that differentiates between WT, heterozygous, and homozygous knock-in clones. A full scan of the gel is included in Supplementary Fig. S1a. The PCR-based genotyping was independently repeated five times with a similar result. **c** Sanger sequencing of the knock-in band to confirm insertion of the reporter at the correct location.

Several protocols for differentiation of OLLCs from hPSCs have been reported^12–14,20–22^. The hOPCs from such differentiating cultures can be purified using antibodies against endogenous O4 or A2B5 surface antigens or against PDGFRα, an OPC-specific surface protein. However, the majority of the O4^+^ cells represent post-mitotic immature oligodendrocytes and the A2B5^+^ cells consist of a heterogeneous population of glial restricted cells and developing neurons^9^. More importantly, since O4 and A2B5 are ganglioside epitopes, there is no easy way to genetically label and track the cells that express these antigens. Since we wanted to monitor the OL differentiation and study them starting at the early progenitor cell stage, we established a platform that enabled us to detect and purify cells expressing *PDGFRα*, a well-established marker for OPCs and pre-OL cells^11,20,23^. We created a reporter hESC line in which the identification-and-purification (IAP) sequence^24^ was engineered to be expressed under the control of *PDGFRα*. The IAP tag consists of a tdTomato fluorescent marker and a mouse cell-surface protein, Thy1.2, separated from each other and from the endogenous *PDGFRα* gene product by the “ribosome-skipping” 2A peptide^24^ (P2A-tdTomato-P2A-Thy1.2) (Fig. 1a). We have previously demonstrated the efficacy of the IAP reporter system for detecting and purifying cells of interest from hESC-derived heterogeneous cell populations, and have shown that P2A functions, as reported^25,26^, to separate the translated gene products so that the endogenous gene remains functionally intact while the fluorescent reporter is cytoplasmic and Thy1.2 is present on the cell membrane^24^.

To generate the PDGFR*α*-P2A-tdTomato-P2A-Thy1.2 hESC reporter (PD-TT), the ESC line WA09 (H9) was engineered to integrate the IAP tag at the 3′-end of the *PDGFRα* gene using CRISPR-based genome editing (Fig. 1a–c). Using transient antibiotic selection^27^, we achieved ~20% homozygous knock-in of the reporter sequence (Supplementary Fig. 1a, b). Sequencing confirmed the absence of mutations at the predicted five most likely off-target genomic locations (Supplementary Fig. 1f), and karyotype analysis performed at different passage numbers showed no abnormality in the early passages (Supplementary Fig. 1c). However, we found isochromosome duplication of the long arm of chromosome 1 in 50% of the clones analyzed by G-banding in the cells after passage #10 (Supplementary Fig. 1c–e). This change is one of the most common karyotypic abnormalities found in hESCs, comprising 10–25% of the total hESCs with aberrations^28,29^.

### Differentiation and purification of PD-TT-derived OPCs

**Fig. 2:**
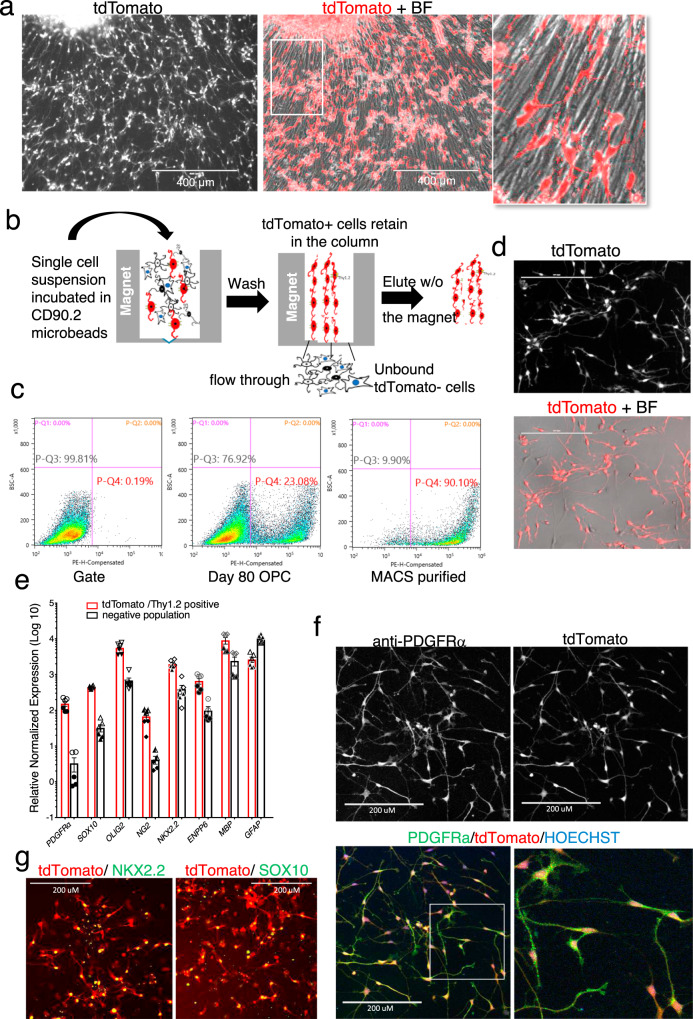
Differentiation and purification of PD-TT hESC reporter cells. **a** Eighty-days-old OPC culture expressing tdTomato driven by the endogenous *PDGFRα* promoter. Differentiation of the PD-TT reporter cells was performed ten times with similar results. Scale bar: 400 μm. **b** Schematic figure of MACS-based immunopurification of reporter cells using anti-Thy1.2 microbeads and a magnetic column. **c** Flow analysis indicating 23.1% of the total cells in differentiating culture are tdTomato^+^ at day 78, which is enriched to 90.1% after MACS purification. PE in *X* axis represents PDGFRα-tdTomato. A gate for flow analysis was set up using non-genome-edited hES cells differentiated to day 95 (left panel). Gating strategy is further detailed in Supplementary Fig. S1j and is also described in the “Methods” section. **d** Majority of the Thy1.2 immunopurified cells are tdTomato^+^ and have the OPC-like bipolar morphology. Scale bar: 200 μm. MACS-based purification and culture of the purified reporter OPCs were performed 10 times with similar result. **e** qPCR analysis shows enrichment of OPC markers in the MACS-based tdTomato enriched population vs the flow through. Two biological and three technical replicates each were used for qPCR analysis. Data are presented as mean ± SEM. Biological and technical replicates are distinguished by solid vs open symbols used for each data point. Source data for the qPCR are provided as a Source Data file. **f–g** Immunohistochemistry demonstrating that the MACS purified tdTomato^+^ cells express PDGFRα (**f**) and the OPC markers NKX2.2 and SOX10 (**g**). Scale bar: 200 μm. Immunohistochemistry was independently repeated twice with similar results.

We differentiated the hESC PD-TT reporter line into OPCs, following the protocol of Douvaras and Fossati^14,30^ (Supplementary Fig. 1g). Analogous to the timing of initial *PDGFRα* mRNA expression, small clusters of tdTomato^+^ cells were visible in the differentiation culture as early as day 8. However, morphologically bipolar, individual, tdTomato^+^ OPCs were not visible until ~day 45, at which time mRNA levels of *PDGFRα* are increased ~700 fold compared to undifferentiated PD-TT cells (Supplementary Fig. 1h, i and Supplementary Movie 1). By day 60, numerous tdTomato^+^ cells were seen migrating out from neurospheres grown on poly-l-ornithine/laminin-coated plates (Supplementary Fig. 1h and Supplementary Movie 2). By day 80, when grown in mitogen-free glial media^30^, ~25% of the total cells in the differentiating cultures are tdTomato^+^ OPCs (Fig. 2a, c).

Since the differentiated reporter OPCs also express the mouse Thy1.2 surface tag, these cells can be immunopurified via anti-Thy1.2 microbeads and magnetic-activated cell sorting (MACS) (Fig. 2b). Of note, the antibodies against mouse Thy1.2 used in the purification are species-specific and do not react against human Thy1^24,31^, and immunopurification using the Thy1.2 surface tag is gentler to the cells and is more amenable to purifying large numbers of cells than fluorescent activated cell sorting (FACS)^24^. Using MACS purification, we routinely obtain an ~90% pure population of tdTomato^+^, PDGFR*α* expressing cells (Fig. 2c, d). When the purified cells are re-plated on a laminin-coated surface, they show bipolar morphology, a characteristic feature of OPCs (Fig. 2d). As they mature, within two weeks in the mitogen withdrawn glial media, tdTomato expression in the cells is reduced and multiple branches and fine cell processes are formed resembling either astrocyte or oligodendrocyte cells (Supplementary Fig. 2a).

### Characterization of the PD-TT cells after OPC differentiation

**Fig. 3:**
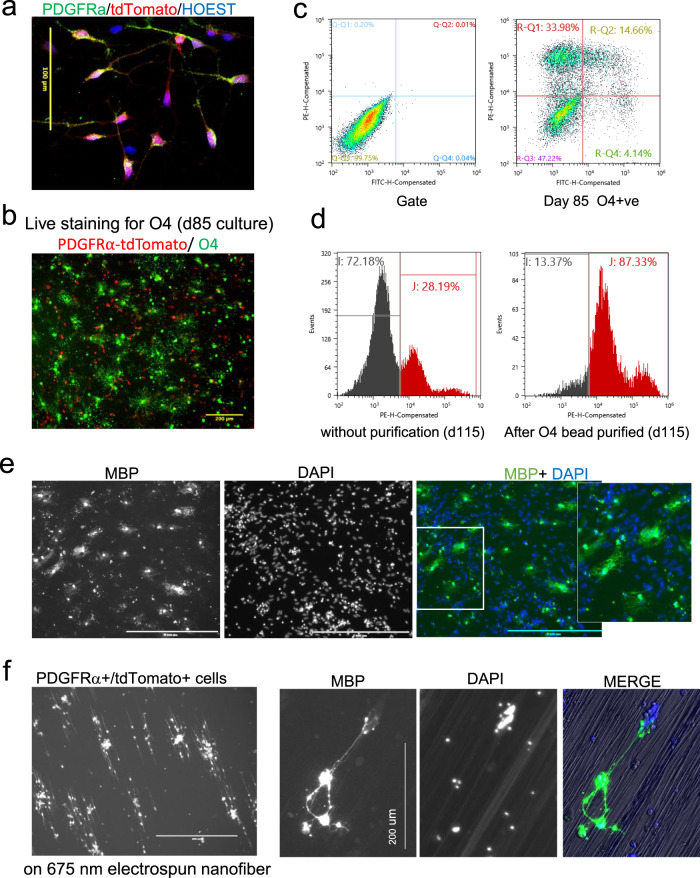
Maturation of reporter OPCs into myelinating oligodendrocytes. **a** Image taken with ×40 objective shows PDGFRα localized to the cell membrane and tdTomato in the cytoplasm. Images for individual channels are included in Supplementary Fig. S3c. Scale bar: 100 μm. Immunohistochemistry with PDGFRα was independently repeated three times with similar results. **b** Live-cell immunofluorescence staining shows that the differentiating reporter OPCs (red) at day 85 express O4 antigen (green). Scale bar: 200 μm. This was independently repeated three times with similar results. **c** Flow-based quantification of the imaged cells shows ~18% O4^+^ cells, of which ~14% are also tdTomato^+^. **d** Flow analysis of tdTomato expression of day 115 before (left) and after (right) MACS-based immunopurification using O4-antibody-conjugated microbeads. After purification, the population was enriched to 87% tdTomato^+^ cells. PE is *X* axis represents PDGFRα-tdTomato. The same gate (left panel of **c**) was used for both **c** and **d** flow analysis. Gating strategy is further detailed in Supplementary Fig. S1j and described in the “Methods” section. **e**, **f** Day 80 Thy1.2 purified population of tdTomato enriched cells were further cultured on PLO-laminin-coated plates (**e**) or on electrospun nanofibers of 675 nm diameter (**f**). **e** Scale bar: 400 μm, **f** left panel, scale bar: 400 μm. Cultures were grown in mitogen-free media for 3 weeks and immunostained with MBP antibody. **e** Independently repeated five times and **f** was independently repeated twice with similar results.

To further characterize the PDGFRα*-*tdTomato-Thy1.2 expressing cells, at day 80 of differentiation, Thy1.2 antibody binding cells were separated from the non-binding cells by MACS purification. Flow analysis showed ~90% of the binding cells were tdTomato^+^ (Fig. 2c). The expression of various OPC markers between the purified and unbound cells was analyzed by quantitative reverse-transcription PCR (qPCR) (Fig. 2e). The qPCR expression data were normalized relative to PD-TT undifferentiated stem cells. The purified cells showed significantly enhanced expression of *PDGFRα* and other OPC markers, including *SOX10, OLIG2, CSPG4* (*NG2)*, and *ENPP6*. Transcripts for *MBP*, an oligodendrocyte marker gene, was enriched in the purified cells compared to the unbound cells whereas *GFAP*, a gene that is expressed at high levels in astrocytes, was partially enriched in the unbound population (Fig. 2e). Immunofluorescence showed that the majority of tdTomato^+^ cells express NKX2.2, OLIG2, and SOX10 (Fig. 2g and Supplementary Fig. S2b), and 80% of the tdTomato^+^ cells are also PDGFR*α*^+^ (Fig. 2f and Supplementary Fig. 3a, b). Furthermore, higher resolution images of cells stained for PDGFR*α* showed that PDGFR*α* localizes to the cell membrane while the tdTomato is cytoplasmic (Fig. 3a and Supplementary Fig. 2c), which indicates that the PDGFR*α* protein product is effectively separated from the reporter proteins.

To further examine whether the differentiating reporter cells are representative of the OL progenitors, we looked at the O4 antigen expression in the tdTomato-expressing cells. At day 85, ~20% of the total cells in a differentiating culture were O4^+^, and 70% of the O4^+^ cells expressed tdTomato (Fig. 3b, c). Only ~30% of the A2B5^+^ cells were tdTomato^+^ at this stage (Supplementary Fig. 3c). When we purified the O4^+^ cells from day 115 differentiating culture using O4 microbeads, we found ~87% overlap between O4^+^ and tdTomato^+^ cells (Fig. 3d). These results are comparable to the amount of overlap between PDGFR*α*^+^ (CD140a^+^) and O4^+^ cells in primary and hPSC-derived human OPCs^11,21^, and shows that expression of the reporter occurs in both early progenitors as well as pre-OL cells and thus can be used to purify cells at various stages of the oligodendrocyte lineage.

When purified tdTomato^+^ cells were plated on either laminin-coated surface or plates containing electrospun nanofibers for another 3 weeks, they continued to differentiate into mature OLs. The OL cultures were stained with an antibody against MBP and MBP^+^ cells displayed the distinct, typical branched morphology of mature OLs (Fig. 3e). The cells cultured on electrospun nanofibers not only developed processes but the processes were aligned along the nanofibers, and appear to myelinate them (Fig. 3f). In addition, the MACS purified cells can be cryopreserved and revived with >80% viability. Upon revival, they maintain the capacity to mature into MBP^+^ OLs (Supplementary Fig. 3d, e). Therefore, these reporter cells provide the flexibility to collect large numbers of cells for studies that require a large amount of material (i.e., biochemical studies, screening applications, etc.).

### Single-cell transcriptome analysis of PDGFRα^+^ reporter OLLCs

**Fig. 4:**
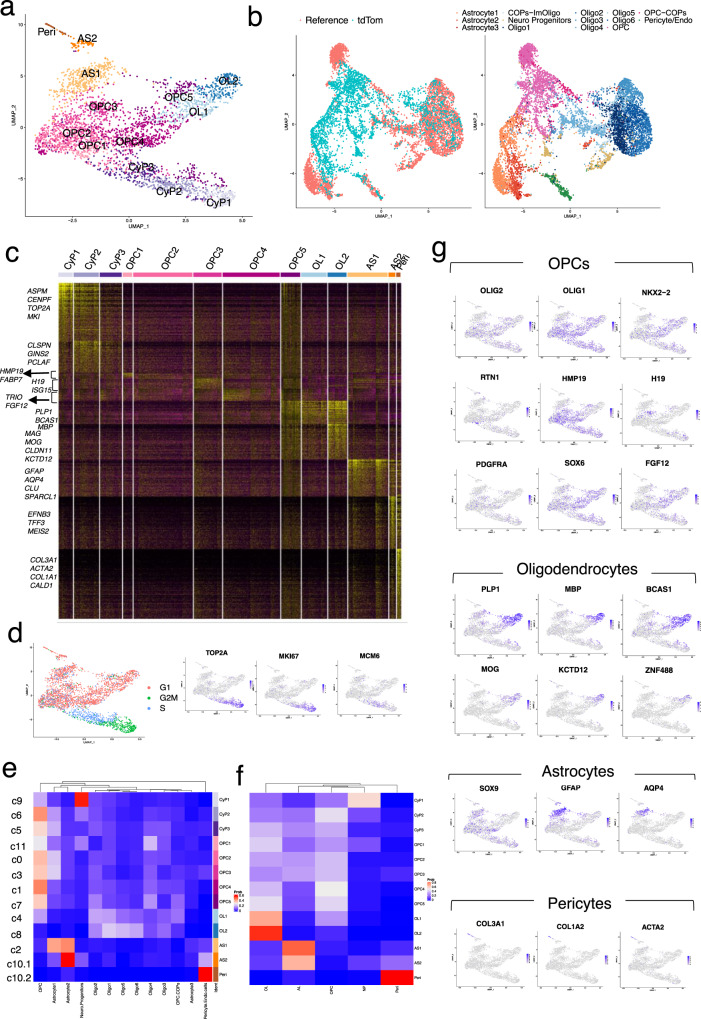
Single-cell transcriptomic analysis of purified OPCs. **a** Unsupervised clustering of the single-cell transcriptional profiles of 3271 purified PDGFRα-tdTomato^+^ cells visualized with as UMAP embedding. Peri pericytes, AS astrocytes, CyP Cycling progenitors, OL oligodendrocytes, NP Neural progenitors. **b** Co-imbedding of our data set (tdTom) with the reference (generated by integrating snRNAseq-based transcriptome from three previously published data sets, Supplementary Fig. S4). Oligo oligodendrocytes, COP committed oligodendrocyte progenitors. **c** Expression heatmap of all the enriched genes from each cluster. Yellow color represents increased expression. **d** Cell-cycle analysis showing cells at G2M, S, and G1 phase. Cell-cycle-related genes are enriched in the cells at G2M an S phase. **e**, **f** Average cell-type label probability per cluster transferred from the combined reference. Rows (left): original cluster numbers (Supplementary Fig. S4b), rows (right): cluster assignment to our data set, column: cell-types from reference data set. **f** Average cell-type label probability assignment after combining six sub-clusters of oligodendrocytes as one OL cluster and 3 astrocyte sub-clusters as one AS cluster. Red color represents higher and blue is used for lower probability. **g** Distribution of various genetic markers among the different clusters presented as an enrichment heatmap. Blue color represents enrichment.

To better understand the gene expression nuances associated with human OPC/OL differentiation, we applied the microfluidic-based “Drop-seq” strategy^16^ to capture the transcriptome of differentiating hOLLCs at the single-cell level. PD-TT reporter cells at three timepoints spanning the in vitro differentiation process (days 77, 89, and 104) were separately MACS purified to ~90% PDGFRα-tdTomato purity, single cells captured, and their transcriptomes determined. After quality control (Supplementary Fig. 4a, Supplementary Data 5, detail in “Methods” section), a combined total of 3271 cells were used for further analysis. Seurat-based unsupervised clustering and visualization with uniform manifold approximation and projection (UMAP) identified 13 distinct cell populations (Fig. 4a, c and Supplementary Fig. 4b).

**Fig. 5:**
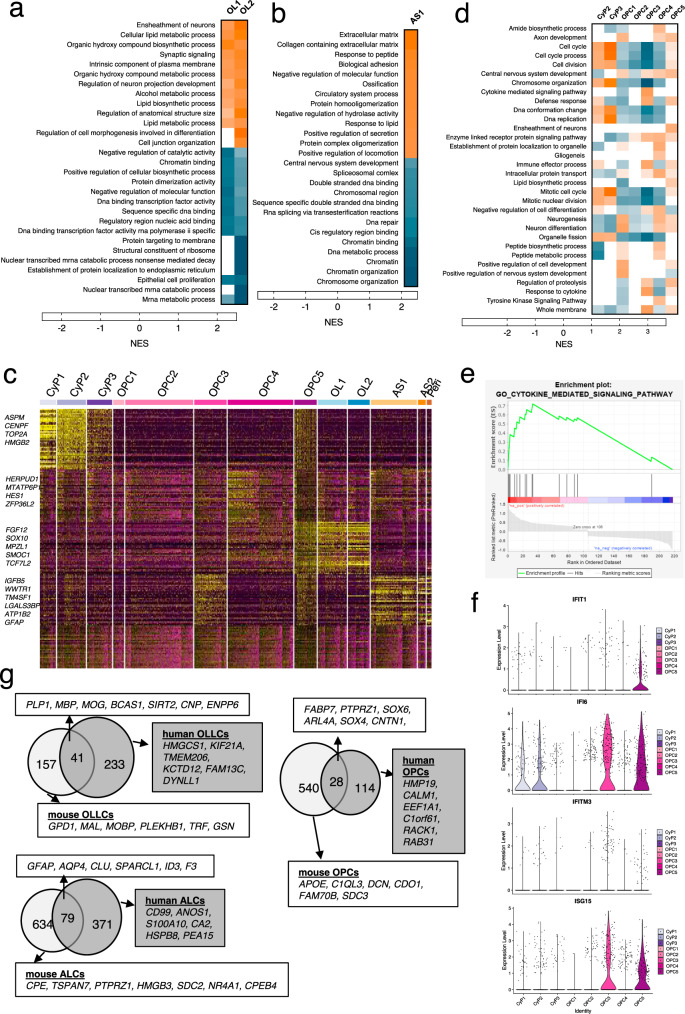
Differential gene expression analysis and GSEA of the scRNA-seq data. Heatmap of GSEA highlighting highly enriched pathways among **a** the OL clusters and **b** the AS1 cluster. **c** Differential gene expression analysis restricted only to the progenitor (CyP2-3 and OPCs1-5) clusters was performed and an expression heatmap of the top 40 enriched genes among the progenitor clusters is presented (**c**). Expression of these enriched genes in the mature cluster is included for visual comparison. **d** The differentially expressed genes from this analysis were used for GSEA and a heatmap of the highly variable pathways among the OPC sub-clusters is presented. Orange color represents upregulated and blue is downregulated pathways. **e**, **f** GSEA enrichment plot for the cytokine-mediated signaling pathway that is enriched in OPC3 (**e**) and violin plots showing expression of genes responsible for the cytokine response in different OPC sub-populations (**f**). **g** Venn diagram depicting the overlap of genes enriched in human vs mouse OLLCs, ALCs, and OPCs, with examples of overlapping and different genes.

We constructed a combined single nuclei RNA-seq (snRNAseq) data sets from adult and fetal human brains^32–34^, and performed integration and label transfer with our data (Fig. 4b and Supplementary Fig. 4b–e). Based on the label prediction and cluster assignment probabilities calculated using the integrated data set, we predicted seven sub-clusters of OPCs, two of OLs, two of astrocytes, and additional clusters for neural precursor cells (NPC) and pericytes in our data set (Fig. 4a, e, f and Supplementary Fig. 4e). Although the three sub-clusters at the bottom of the UMAP expressed OPC markers, they had enriched expression of cell-cycle-related genes (*TOP2A, PCNA, MKI67,* and *MCM6)* and they were in the G2M/S phase of the cell cycle (Fig. 4d and Supplementary Figs. 4h,5a); therefore, they were labeled as cycling progenitors^35^. More mature cells branch out to form more discrete clusters and seem to represent astrocytes and oligodendrocytes that are in the G1 phase of the cell cycle (Fig. 4a, d). Mature OL markers such as *MAG, MOG*, and *ZNF488* were more enriched in cluster OL2 (Fig. 4g, Table 1 and Supplementary Fig. 4h), suggesting that it represents a more mature OL population than cluster OL1. Notably, the OL population in our hESC-derived cells share similarity with all 6 of the OL sub-populations (Fig. 4b, e and Supplementary Fig. 4e–g)^32^. Gene set enrichment analysis (GSEA) showed enrichment of lipid biosynthesis, ensheathment of neurons, and synaptic signaling in both OL populations whereas extracellular matrix, biological adhesion, and protein homooligomerization-related genes were enriched in the AS populations (Fig. 5a, b and Supplementary Data 3.).

**Table 1:** Top 40 enriched genes per cluster ranked by fold change.

Cycling progenitors			OPCs					Oligodendrocytes			
CyP1	CyP2	CyP3	OPC1	OPC2^a^	OPC3	OPC4	OPC5	OL1	OL2	AS1	Peri
ASPM	HIST1H4C	PCLAF	RTN1	HMP19	IFI6	TRIO	PLP1	BCAS1	MBP	GFAP	COL3A1
CENPF	PCLAF	CLSPN	CRABP1	FABP7	H19	HES1	BCAS1	MIR219A2	PLP1	CLU	LGALS1
UBE2C	TOP2A	GINS2	STMN2	HIP1	ISG15	FGF12	IFI6	SIRT2	CNP	AQP4	COL1A1
PTTG1	HMGB2	TYMS	ELAVL4	NRXN1	IGFBP5	PHLDA1	SIRT2	KIF21A	RP11-89N17	SPARCL1	TAGLN
TOP2A	NUSAP1	ORC6	NNAT	STMN4	B2M	HEY1	ARHGAP5	PLP1	CLDN11	ID3	ELN
CCNB1	MKI67	CENPF	SOX4	SCG3	HLA-B	SOX6	FAM13C	WASF1	PTGDS	AGT	SPARC
CENPE	TUBA1B	HMGN2	DCX	KCND2	HLA-A	ARL4A	SLC44A1	GPC3	UGT8	S100A10	ACTA2
NUF2	CDK1	SMC2	SOX11	NKAIN4	HS3ST1	ZFP36L2	RP11-89N17	PRDX1	BCAS1	CD99	COL1A2
HMGB2	SMC4	DUT	MIAT	OLIG1	HLA-C	EGR1	PRDX1	UGT8	KCTD12	EDNRB	FBLN1
NUSAP1	PCNA	MCM7	NREP	CCND2	CD9	RPS2P5	WASF1	SLC44A1	MIR219A2	NMB	BGN
TPX2	RRM2	TMSB15A	ETV1	CALM1	FOS	MAP2	CENPJ	FAM13C	SEMA4D	MGST1	MGP
MKI67	HIST1H1D	MCM4	HMP19	C1orf61	WWTR1	RPL39	FERMT1	CNP	RGS16	AHCYL1	CALD1
CKS2	TPX2	MAD2L1	PEG10	RAB31	IFI16	KHDRBS3	FYN	EPB41L2	SIRT2	TGFB2	SERPINH1
PRC1	HMGN2	H2AFZ	NRXN1	REV3L	SERPINE2	RP4-765C7	CNP	RAB33A	NFASC	CRYAB	TPM4
KPNA2	CENPF	TUBA1B	TAGLN3	ID2	TRIB2	NKX2-2	MBP	SGK1	APLP1	VIM	COL4A1
SGO2	SMC2	RPA2	FTX	PTPRZ1	PMP2	PLPPR1	RNF13	CADM2	HMGCS1	HSPB8	S100A11
NEK2	BIRC5	TUBB	CD24	DBI	RBP1	RPL13P12	KIF21A	SMOC1	CDK18	EZR	TMSB4X
KIF14	ESCO2	PCNA	TERF2IP	RACK1	BAALC	LIMA1	CADM2	ARHGAP5	SLC44A1	SPARC	GPC3
BIRC5	TYMS	RANBP1	PIK3R1		CNTN1	HES5	RND2	FERMT1	DLG1	DTNA	DCN
SMC4	CENPU	NASP	CEP170		NUPL2	GABPB1-AS1	TNR	DYNLL1	CHN2	FABP5	ITGB1
AURKA	MAD2L1	TOP2A	NFIB		SAT1	MTATP6P1	ATCAY	NFASC	FRMD4A	CA2	CRABP2
ARL6IP1	PRC1	SNRPB	KDM5B		PLLP	HIST3H2A	UGT8	FYN	MOG	ID1	COL4A2
CDK1	KNL1	ARL6IP1	HES6		WSCD1	RPL18A	MIR219A2	GALNT13	KIF21A	ID2	OGN
DLGAP5	ATAD2	SUPT16H	FNBP1L		C1orf21	RPL36A	MOG	TMEM206	ARHGAP5	ANOS1	FN1
MAD2L1	ZWINT	HMGB1	CRMP1		CARS	EPN2	GNB4	CENPJ	CENPJ	PEA15	ASPN
FAM64A	DUT	RRM1	TCF12		CCND1	TAOK3	MYO5A	GPR17	RAB33A	F3	MYL9
CDC20	H2AFZ	SRSF2	DLL3		FABP7	COL11A1	EPB41L2	SEMA6D	TNR	DCLK1	IGFBP7
GTSE1	HIST1H1E	NUCKS1	PKIA		MTSS1	CCND1	SMOC1	FRMD4B	ENPP6	CNN3	SELENOM
CCNB2	UBE2C	SMC3	TTC3		DBI	GAS5	SLC25A5	HIPK2	TM7SF3	PSRC1	TMSB10
CDKN3	NDC80	CKS1B	STMN4		NDFIP1	STMN2	MOB3B	SLC25A5	RND2	ATP1B2	PCOLCE
CKAP2	TUBB	GNG4	MAP1B		GPR37L1	ETV1	CRB1	ATCAY	CTD-2636A23	CST3	LUM
KNL1	MCM4	SMC4	FAM110B		ATP1B2	TMEFF2	HIPK2	E2F3	FAM13C	ID4	COL6A3
CCNA2	DHFR	AP2S1	STMN1		MALSU1	GRIA2	ERBB3	SGCD	SGCD	TNC	PALLD
KIF4A	KIF15	CACYBP	ZNF292		HMP19	RP11-343H5	MPZL1	TNR	PRDX1	B2M	LAPTM4A
CKS1B	CLSPN	HMGB3	SLC38A1		RAB31	RPSAP58	PKP4	APOD	GOLIM4	CCDC80	SSR2
HMGB3	ANP32E	BZW2	TCF4		CD59	CA10	NFASC	SOX10	TRIM2	IGFBP7	ACTN1
ANP32E	MIS18BP1	PA2G4	MAP2		HIP1	AC007969	GPR17	TCF7L2	ARHGAP5-AS1	RGMA	WFDC1
TUBB4B	NUF2	NOP56	HDAC2		PHLDA1	RPS29	RAB33A	ENPP6	MAG	HOPX	AKAP12
KIF20B	TMSB15A	EXOSC8	MLLT11		TRIM9	TRAF4	CHN2	SOX2-OT	FYN	PON2	TPM2
NDC80	PTTG1	HNRNPD	KIFAP3		RGS16	DANCR	TNS3	GRIA2	FBXO32	SPON1	CDH11

The entire list of genes can be found in Supplementary Data 1.

*OPC* oligodendrocyte progenitor cells, *AS* astrocytes, *Peri* pericytes.

^a^Only 19 enriched genes were identified for OPC2.

A relatively isolated, mature cluster at the top of the UMAP contained AS and pericyte sub-clusters (Fig. 4a). The smaller sub-cluster expressed a number of pericyte marker genes (*COL1A1, COL1A2, COL3A1, ATAC2)*^36,37^ that are responsible for collagen and extracellular matrix formation and organization, organ morphogenesis, and cardiovascular development (Fig. 4g, Table 1 and Supplementary Fig. 5c, Supplementary Data 3). This group of cells likely represent the VLMC-pericyte cells that are reported to arise from PDGFRα^+^ precursors^38^. The other sub-cluster shared similarity to a subgroup of mature astrocytes (Fig. 4e, f) marked by the enriched expression of *GFAP, EFNB3, and TFF3* (Fig. 4g and Supplementary Data 1).

### scRNA-seq provides insight into the PDGFRα-reporter OPC sub-populations

Differential gene expression and GSEA within the OPC sub-clusters revealed a number of differences between them (Fig. 5c, d and Supplementary Data 2, 3). OPC1 shared similarity to a previously reported oligo 4 sub-cluster^32^ (Fig. 4e), but it also expressed a number of genes (*STMN2, NREP, MAP1B, SOX11*) involved in neurogenesis, and neuron differentiation (Table 1), which suggests that the cells in this OPC sub-population may have the capacity to differentiate into neurons. OPC3 was enriched for cytokine-mediated signaling pathways, immune response, and defense response genes (*IFI6, ISG15, IFIT1, HLA-A, B and C*), and likely represents a sub-population of OPCs that can respond to cytokines^39–43^ (Fig. 5d–f and Supplementary Fig. 5d, e). OPC4 showed enriched expression of gliogenesis (*SOX6, SIRT2, SOX10)* as well as neurogenesis (*HEY1, TRO, HIS1*)-related genes. Receptor protein tyrosine kinase signaling pathway, amide biosynthetic process, and peptide biosynthetic process-related gene sets were also enriched (*HES1, DDIT4, FGF12, ZFP36L2, RPL39*) in this cell population (Fig. 5d and Table 1). The OPC5 sub-cluster had enriched expression of a number of OL markers and biological functions such as lipid biosynthesis and ensheathment of neurons that are similar to that seen with the OL clusters. The cells in this sub-cluster were also in S phase and are enriched for cell cycle and cell division gene sets (Fig. 4d and Table 1), which indicates that they might be a unique group of proliferative cells at an intermediate oligodendrocyte state. The differential gene expression analysis showed that OPC3 and OPC4 shared a number of enriched genes with astrocytes and OLs, respectively, which indicates that these sub-populations of OPCs might already be inclined towards either astrocyte or OL lineages (Fig. 5c).

Since the CyP1 and CyP2 expressed OPC marker genes and also showed similarity to the OPCs by cluster assignment probability (Fig. 4e, f), they were included in the analysis as OPC sub-clusters. However, mitotic processes and cell cycle events were dominant in these groups of cells (Supplementary Fig. 5b). OPC2, on the other hand, expressed OPC markers, and although it expressed only a limited number of enriched genes, a large set of genes were downregulated compared to the other OPCs (Fig. 5d and Table 1).

### Potential of PDGFRα^+^ precursor cells to differentiate into cells expressing astrocyte markers

Of all the single cells analyzed, 901 (27.5%) expressed *GFAP* and 733 (22.4%) expressed *MBP*, markers for astrocyte lineage cells (ALCs) and OLLCs, respectively (Supplementary Fig. 5f). The number of OPCs decrease while the number of OL and AS cells increase over time as they mature (Supplementary Fig. 5f–h), which supports a previous report that PDGFR*α*^+^ human OPCs have the potential to mature into both astrocytes and oligodendrocytes in vitro^11^. We also analyzed the tdTomato^+^ cells from days 77, 89, and 104 separately (Supplementary Fig. 6a–c). Cells from each timepoint formed distinct clusters with OPC, OL, and AS enriched populations. Although cells from later timepoints are enriched in the mature clusters, cells from each timepoint were present in all other clusters as well (Supplementary Fig. 6e), which confirms that purified hESC-derived OLLCs, even at the same in vitro differentiation timepoint, are temporally heterogeneous in terms of their degree of differentiation.

### Validation of findings, particularly related to the astrogenic potential of OPCs and the sub-populations of OPCs

**Fig. 6:**
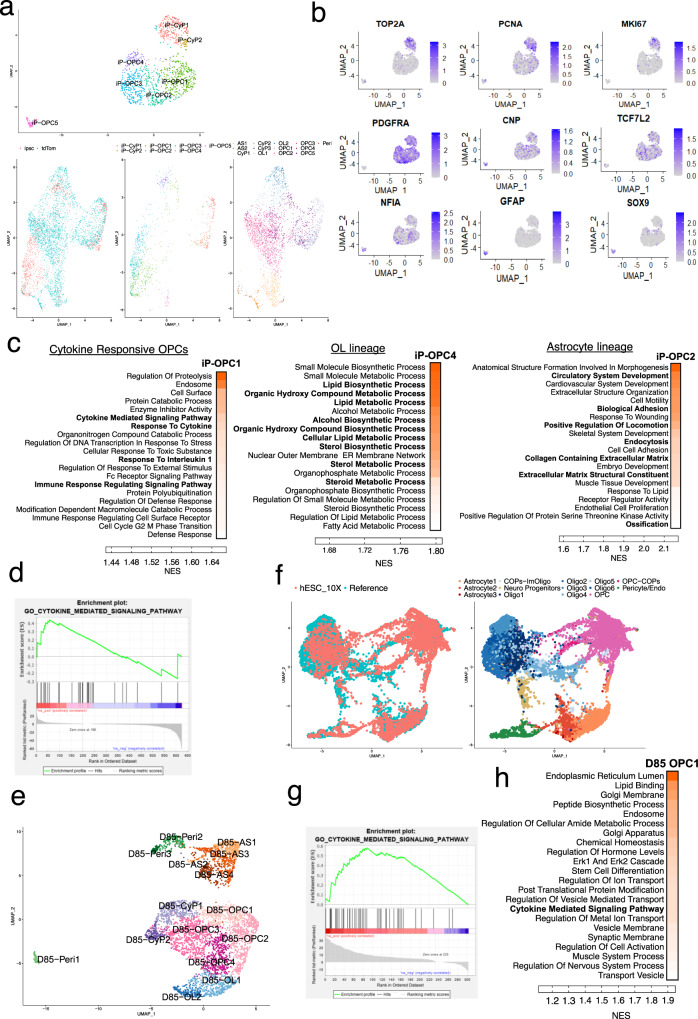
Single-cell transcriptomics and Go term analysis of hiPSC-derived PDGFRα^+^ OPCs and an independent batch of purified PDTT-OPCs. **a** Unsupervised clustering of the single-cell transcriptional profiles of FACS purified PDGFRα^+^ cells derived from hiPSCs. Visualization with a UMAP embedding divides the single cells into seven clusters. On the lower panel, co-imbedding of the hiPSC-derived data set (ipsc) with the hESC-derived PDGFR-tdTom data set from Fig. 4 shows the two data sets have overlapping cell populations. **b** Distribution of various genetic markers among the different clusters presented as an enrichment heatmap. All the differentially expressed genes are listed in Supplementary Data 7**. c** Heatmaps of GSEA show cluster-specific enrichment of OL and AS-related pathways and highlights sub-populations of OPCs that are cytokine-responsive. Detailed GSEA for all the clusters is listed in Supplementary Data 7. **d** GSEA enrichment plot for cytokine-mediated signaling pathway that is enriched in iP-OPC1. **e** Unsupervised clustering of the single-cell transcriptional profiles of D85 purified PDGFRα-tdTomato^+^ cells from an independent batch of differentiation. Peri pericytes, AS astrocytes, CyP Cycling progenitors, OL oligodendrocytes. **f** Co-imbedding of the D85 data set (hESC_10X) with the reference (generated by integrating snRNAseq-based transcriptome from three previously published data sets, Supplementary Fig. S4). Oligo oligodendrocytes, COP committed oligodendrocyte progenitors. **g** GSEA enrichment plot for cytokine-mediated signaling pathway that is enriched in D85-OPC1. **h** Heatmaps of GSEA for OPC sub-cluster (D85-OPC1) highlighting cytokine-mediated signaling pathway. Detailed GSEA for all the clusters is listed in Supplementary Data 6. **c**, **h** darker orange indicates higher enrichment.

To confirm the reproducibility of our single-cell transcriptomic and other findings on the astrogenic potential of hOPCs and on the sub-populations of hOPCs, we further analyzed the single-cell transcriptomes of additional stem cell-derived hOPCs. This validation analysis included: (1) an independent non-reporter hiPSC line differentiated into OPCs (iP-OPCs) and sampled by a different laboratory group, and (2) an independent batch of hOLLCs differentiated from the PD-TT reporter line (D85-OLLCs) (Fig. 6 and Supplementary Figs. 7–9). Sixty-day-old iP-OPCs were FACS purified using PDGFRα antibody (Supplementary Fig. 7a) and day 85 PD-TT reporter OLLCs were MACS purified with thy1.2 microbeads prior to single-cell capture using 10× platform. Although at the D60 timepoint, as expected, there were only a few cells that expressed mature OL and astrocyte markers, the co-imbedding of the hiPSC-OPC data set with the OLLC data set from Fig. 4 indicated that the iP-OPCs were fully consistent with the trajectories we defined with the PD-TT-derived reporter OLLCs (Fig. 6a and Supplementary Fig. 7b). Additionally, expression of cell-cycle-related genes *(*e.g., *PCNA, TOP2A, MKI67)*, OL-related genes (*CNP* and *TCF7L2*), as well as TFs are known to drive astrocyte differentiation (*SOX9*, *NFIA*, and *NR2F1*), were present in these D60 cells (Fig. 6b and Supplementary Fig. 7c). GSEA analysis on the iP-OPC data set also showed cluster-specific enrichment of OL and AS-related pathways and highlighted sub-populations of OPCs that are cytokine-responsive and OPCs that are inclined to either OL or AS lineages (Fig. 6c and Supplementary Fig. 8, Supplementary Data 7). Additionally, the D85-OLLCs integrated well with the reference data set from Supplementary Fig. 4c, d (Fig. 6e, f). The OL, AS, pericyte, cycling progenitor cell populations, and different OPC sub-populations including the cytokine-responsive OPC sub-populations, were present in the D85 data set as well (Fig. 6e–h and Supplementary Fig. 9a–e, Supplementary Data 6).

In addition, we also performed scRNA-seq analysis of day 89 PD-TT reporter OPCs purified by an independent method, using O4-antibody-conjugated microbeads. Similar to the *PDGFRα* expressing cells, a sub-population of the O4^+^ cells also express astrocyte markers (Supplementary Fig. 6d).

### ScRNA-seq reveals distinct cell type-enriched genes, some of which appear to be species-specific

We next examined other highly enriched and differentially expressed genes from each of the clusters illustrated in the UMAP plot (Fig. 4a and Table 1). The majority of the differentially expressed genes from the OLLCs and ALCs populations are consistent with the previous reports^3,44^. Genes that have been reported to be specifically enriched in human OLs and astrocytes but not expressed in mouse OLs or astrocytes, such as *APCDD1*, *HMGCS1, PMP2*, and *WIF1*^44–46^, were also enriched in the respective clusters as expected. We assessed the overlap of genes enriched in our hESC-derived OLLC, ALC, and OPCs to that of mouse OLLCs, ALCs, and OPC-enriched genes^3^. Although numerous genes including known marker genes overlapped between the two data sets, we noted several differences (Fig. 5g, Supplementary Fig. 9f, Source Data). We found OL-specific enrichment of several genes (e.g., *KCTD12*, *SLC7A14, HMGCS1, SPOCK1, FAM13C*, *FAM131C, TMEM206*, and *KIF21A)* in our system that contrast with previous publications. For example, *KCTD12*, *TMEM206*, *FAM131C*, *FRMD4B,* and *APCDD1* were all enriched in human OLs in our system, but in the mouse, based on bulk RNA-seq of purified CNS cells, *KCTD12* and *TMEM206* are enriched in microglia; *FAM131C* is enriched in neurons, and *FRMD4B* and *APCDD1* expression is specific to endothelial cells^3^.

We additionally identified a number of differentially expressed primary microRNAs (pri-miRNAs) in our scRNA-seq data (Supplementary Fig. 6f). For example, miR219-A2 was enriched in the OL clusters while miR100HG and miR99AHG were enriched in the astrocyte clusters. These differentially expressed pri-mRNAs are potentially involved in regulating the fate of an OPC to become either an OL or an astrocyte.

### Pathway enrichment analysis reveals pathways associated with OPC sub-populations, and oligodendrocyte and astrocyte differentiation

**Fig. 7:**
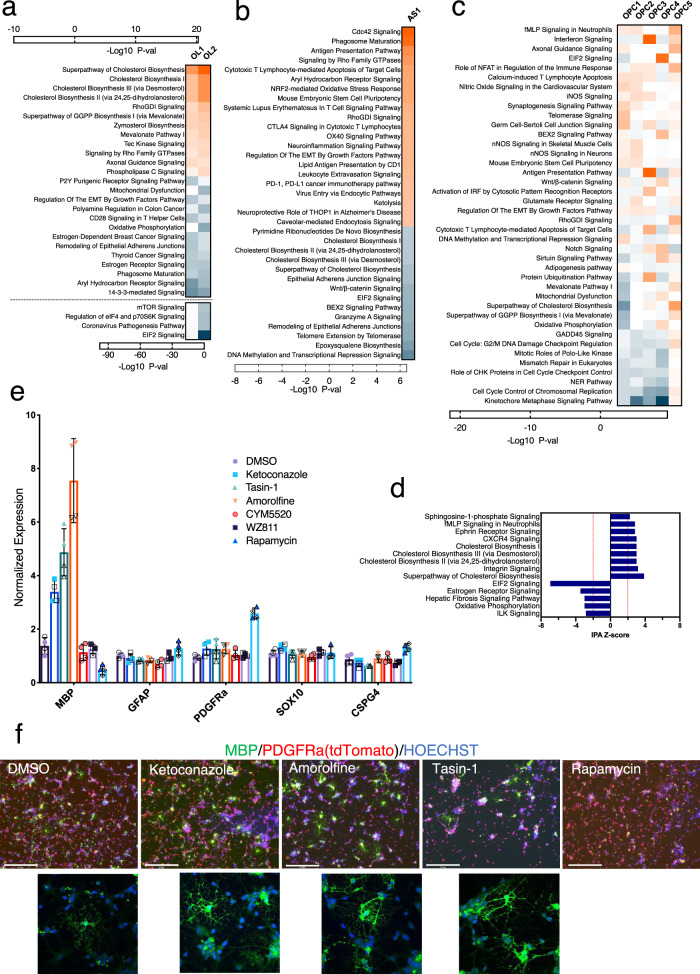
Pathway analysis of the differentially expressed genes. Top enriched pathways implicated for **a** oligodendrocytes **b** astrocyte, and **c** OPC sub-clusters presented as a heatmap. **a** Pathways in the OL clusters with very low *P*-values are presented separately under the dotted line. **c** Gene sets from the differential gene expression analysis restricted only to the progenitor clusters (CyP2-3 and OPCs1-5) were used for this IPA. Colors indicate significance based on −log10 *P*-value. Orange color represents upregulated and blue is downregulated pathways. **d** Pathways that are significantly differentially modulated in the mature OL cluster. *Z*-score for each pathway was calculated by IPA. **e**, **f** Reporter hOPCs purified at day 85 of differentiation were cultured in the presence of DMSO or compounds that inhibit CYP51A1 (ketoconazole), TM7SF2 (amorolfine), EBP (tasin-1), S1P (CYM5520), CRCX4 (WZ811), or mTOR (rapamycin) signaling pathways. **e** The treated cells were tested by qPCR to assess the effect on hOPC differentiation to OL. Two technical and two biologicals replicates each were used for the qPCR. Biological and technical replicates are distinguished by solid vs open symbols. Data are presented as mean ± SD. Source data are provided as a Source Data file. **f** Immunostaining of the cells treated with different compounds shows stronger MBP staining and more MBP^+^ cells in the samples treated with CBP inhibitors. Scale bar: 275 μm. Immunostaining was independently repeated twice with similar results.

In order to get further insight into the molecular pathways that define OPCs, OLLCs, or ALCs sub-populations, we performed pathway enrichment analyses on differentially expressed genes from each cluster using Ingenuity Pathway Analysis (IPA). In the mature OL2 cluster, a number of pathways including CXCR4, Sphingosine-1-phosphate (S1P), and integrin signaling pathways (ISP), which are known to be important for OPC maturation, oligodendrocyte survival, and myelination, were upregulated^47–49^, and EIF2, ILK, and Estrogen receptor signaling were downregulated, with strong *z*-scores (Fig. 7d). Numerous pathways with P-values denoting significance were also identified for the OPC sub-clusters, and OL and AS clusters (Fig. 7a–c and Supplementary Fig. 10a, b, Supplementary Data 4). The cholesterol biosynthesis pathways (CBPs) and the cholesterol biosynthesis intermediate (CBI) such as mevalonate and zymosterol signaling, known to be involved in OL differentiation and myelination^50^, were enriched in both the OL clusters. The mTOR, eIF4 and P70S6K, pathways were differentially downregulated between the OL1 and more mature OL2 sub-clusters (Fig. 7a). The Cdc42 and Rho-family GTPases signaling, Phagosome maturation and Caveolar‐mediated endocytosis signaling, and Ketolysis, which are implicated in astrocyte functions^50–54^, were enriched while the CBPs, Epoxysqualene, and Wnt/b-catenin signaling pathways were comparatively downregulated in the astrocyte cluster (Fig. 7b). EIF2 signaling demonstrated a wave-like pattern. It was downregulated in the early progenitors, upregulated in OPCs, and downregulated again in the OLs and AS populations (Fig. 7a–c and Supplementary Fig. 10a, b).

We also performed IPA on the OPC sub-clusters. In one analysis we looked at the genes differentially expressed between the OPC sub-clusters vs all the other clusters. In another comparison, we focused on the genes differentially expressed between the individual OPC sub-clusters. Both analyses (Fig. 7c and Supplementary Fig. 10b) showed enrichment of antigen presentation and interferon signaling in OPC3. The Cdc42 and Caveolar-mediated endocytosis signaling and phagosome maturation were also upregulated in this sub-cluster, which further supports that the cells in this sub-cluster are inclined towards astrocyte lineage. EIF2, BEX2, mTOR, Notch, Sirtuin, and Wnt signaling were upregulated and the antigen presentation and interferon signaling were downregulated in OPC4. A number of cholesterol and GGPP biosynthesis-related pathways, that are implicated in OL maturation, were enriched in the OPC5. Nitric oxide and iNOS signaling, and nNOS in neurons, were comparatively upregulated in OPC1 and 2 cells. We also found upregulation of the long non-coding RNA (lncRNA)-HOTAIR pathway, which is known to modulate PI3K/AKT/mTOR signaling, as well as enrichment of the mTOR signaling in the OPC1 sub-cluster. As expected, the cycling progenitors were enriched for the cell-cycling-related pathways and NER, BER, and ATM signaling, which were downregulated in all but OPC5 (Supplementary Fig. 10a). The pathway enrichment analysis further supports the result from the GSEA that the OPC sub-populations are functionally diverse and developmentally heterogeneous.

Of potential interest, we observed enrichment of genes related to coronavirus pathogenesis pathway (*CCND1*, *EEF1A1, RPS29, FOS, STAT1, E2F3*) in the OPC3-5 (most significantly in OPC4) sub-clusters (Supplementary Fig. 10b). This finding suggests the possibility that the SARS-CoV-2 virus, which is causing the current COVID-19 pandemic, may have the capacity to infect CNS progenitor cells. This possibility is consistent with the recent report that iPSC-derived BrainSpheres can be infected with SARS-CoV-2, and that the infected BrainSpheres can support viral replication^55^.

To confirm the biological relevance of a number of above-described, bioinformatically implicated, pathways in hOPC to hOL maturation, we experimentally tested the consequences of pharmacologically inhibiting their activity in developing hOPCs. Similar to a recent report studying murine OL differentiation^50^, inhibition of CYP51A1, TM7SF2, and EBP, which are associated with the CBP, increased expression of *MBP* transcripts in our human system (Fig. 7e). Immunostaining confirmed stronger MBP expression and more MBP^+^ cells in these samples (Fig. 7f). Inhibition of mTOR signaling by rapamycin reduced the expression *MBP* and increased the expression of the OPC markers *PDGFRa* and *CSPG4* (*NG2*), which indicates that the mTOR signaling, although downregulated in the OLs, is still essential for the maturation of hOL from hOPCs. However, targeting the S1P and CXCR4 pathways, at least with the compounds we tested, did not show any significant effect on hOL differentiation/maturation.

### Pseudotemporal trajectory analysis further defines the bipotential nature of PDGFRα^+^ cells

**Fig. 8:**
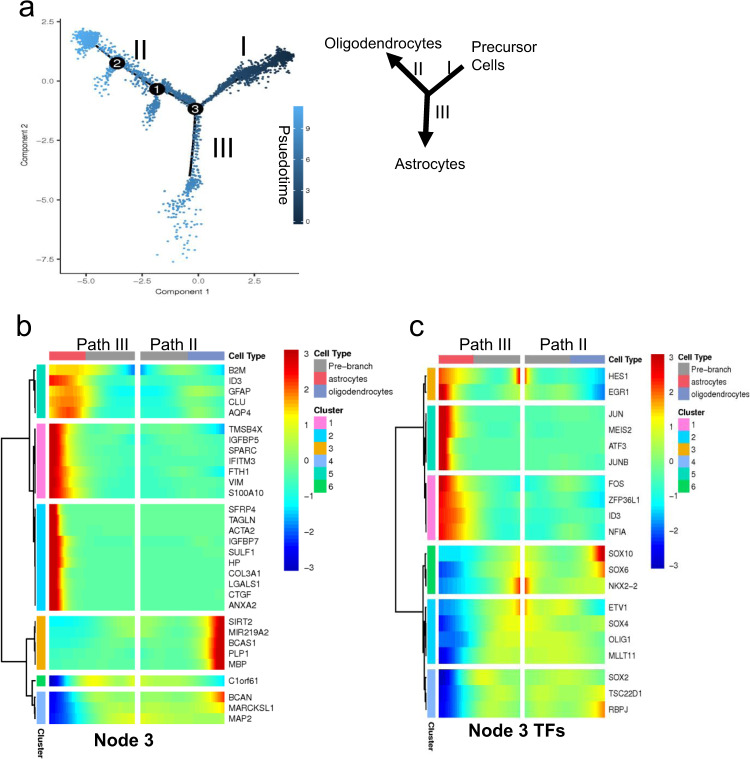
Pseudotemporal trajectory analysis of the single-cell transcriptomes of the hOPCs. **a** Developmental trajectories of the purified reporter OPCs generated, using Monocole-2, demonstrate two prominent paths for the OPCs. Darker colored dots (on Path I) represent developmentally younger cells and the lighter colored dots (on Paths II and III) represent more mature cells. **b**, **c** Kinetic heatmap generated using BEAM regression model capturing the most differentially expressed genes (**b**) and transcription factors (**c**) between Paths II (oligodendrocytes) and III (astrocytes). The genes are hierarchically clustered based on their expression pattern. Red color represents upregulation and blue is used for downregulation.

We performed monocle-based pseudotime analysis on our scRNA-seq data to create a developmental trajectory tracing the lineage specification of PDGFRα^+^ cells as they mature. Analysis of the pseudotemporal trajectory presented two prominent paths for the precursor cells, suggesting that the PDGFR*α*^+^ cells can follow two distinct cell lineages (Fig. 8a). We examined the highly differentially expressed genes and transcription factors (TFs) between the two trajectories and identified path II as OLLCs and path III as ALCs using the Branch Expression Analysis Modeling (BEAM) regression model (Fig. 8b, c).

**Fig. 9:**
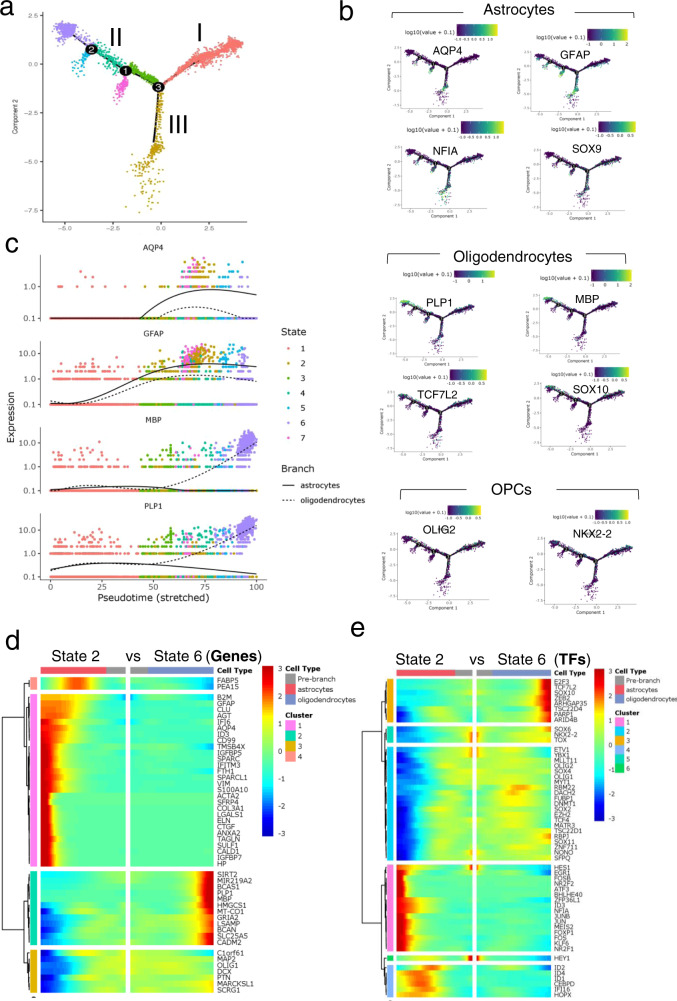
Pseudotemporal trajectory of differentiation based on single-cell transcriptomes. **a** Ordering cells along the trajectory divides the population into seven different states. State 1 cells are OPCs, state 2 cells are astrocytes, and state 6 cells are oligodendrocytes. **b** Expression of OL, astrocyte, and OPC markers within the trajectory further confirms Path III as the astrocyte and Path II as oligodendrocytes lineage cells. Astrocyte makers (*GFAP, AQP4, SOX9, NFIA*) are highly enriched in Path III and in the smaller branches that emerge from path II. OLLC markers (*SOX10, MBP, PLP, TCF7L2*) are enriched in Path II, but not in the smaller branches. Green/yellow represents enrichment. **c** Pseudotemporal expression pattern of OL and astrocyte genes show that the OL genes, *MBP* and *PLP1*, have similar kinetic trend and both are enriched in state 6 cells. The astrocyte marker *GFAP* is modeled to be expressed earlier than *AQP4*, although both are enriched in state 2 cells. **d**, **e** Kinetic heatmap of the most differentially expressed non-transcription factor (**d**) and transcription factor genes (**e**) between state 6 (oligodendrocyte cells) and state 2 (astrocyte cells) identified by BEAM and clustered based on their expression pattern. Red color represents upregulation and blue is used for downregulation.

The monocle-based developmental trajectory analysis defined seven distinct cellular states (Fig. 9a). Cells in state 1 were precursor cells, state 2 was composed of astrocyte cells, and state 6 consisted of oligodendrocyte cells. Analysis of the pseudotemporal expression pattern of the OL and astrocyte genes indicates that the OL genes *MBP* and *PLP1* have similar kinetic trends and both are enriched in state 6 cells, while the astrocyte markers *GFAP* and *AQP4* are enriched in state 2 cells (Fig. 9c). State 5 and 7 cells diverged from the OL trajectory, and were interestingly enriched for astrocyte markers rather than OL markers, and share a stronger correlation with the state 2 astrocyte cells (Fig. 9b and Supplementary Fig. 11a). We also performed pseudotime analysis on the day 89 O4^+^ cells. Similar to the purified PDGFRα-tdTomato^+^ OPCs, the O4^+^ cells also showed two potential lineages as indicated by OL vs astrocyte trajectories and the kinetics of gene expression within the trajectories (Supplementary Fig. 11d–f).

We also examined the most differentially expressed genes and transcription factors at nodes 1, 2 and 3, and in cells at states 2 vs state 6, using the BEAM regression model (Fig. 9d, e and Supplementary Fig. 12a, b). This analysis identified numerous differentially expressed genes of potential interest. In addition to TFs previously implicated in OPC/OL/astrocyte differentiation, such as *SOX10 and TCF7L2*, this analysis implicated a number of previously less characterized factors in OPC/OL/AS differentiation, including *ZEB2, TSC22D4, ARID4B, PARP1, E2F3, and ARHGAP35*, which were significantly enriched in OLs, and *HES1, FOSB, NFIA, NR2F1*, and *ZFP36L1*, which were enriched in astrocytes. Numerous TFs, such as *SOX4, SOX11, MLLT11, RBM22, ZNF711, EZH2*, and *DACH2*, were slightly upregulated in OLs but highly downregulated in ALCs (Figs. 8b, c and 9d, e). A number of these genes are already enriched in day 60 hiPSC-derived OPCs (Fig. 6b, Supplementary Fig. 7d). Furthermore, each path of the trajectory consisted of cells from all three (days 77, 89, and 104) timepoints (Supplementary Fig. 12c), which further supports that PDGFR*α*^+^ cells are transcriptionally and developmentally heterogeneous, with some cells, even at the earliest timepoint tested (day 77), being already relatively mature.

## Discussion

Recent advances in stem cell biology and differentiation methodology have led to the development of protocols for the generation of hOLLCs from hPSCs^12^, and hold the promise of aiding in the development of remyelination-based approaches for the treatment of MS and other demyelinating diseases. The ability to promote remyelination is also highly relevant for ongoing work in regenerative medicine, such as efforts to promote optic nerve regeneration^56^. In current human stem cell differentiation protocols, hOPCs generally make up <50% of the resulting differentiated cell population^12,20,21^, often requiring FACS-based purification for downstream studies. Here, in order to develop a simple and efficient method for obtaining highly purified populations of PDGFR∝ expressing hOLLCs, we used CRISPR-based genome editing to introduce an IAP (P2A-tdTomato-P2A-thy1.2) tag into the endogenous *PDGFR∝* locus. The resulting reporter cell line allows for optimization, scalable differentiation, and purification of hOLLCs (>90% PDGFRα*/*tdTomato-expressing cells) at different stages of differentiation. The cells purified by this method maintain high survival, competence, and the capacity to mature into MBP^+^ OLs even after long-term cryopreservation. This reporter system provides a useful resource and a powerful tool for optimizing new, more efficient differentiation protocols, enabling the easy quantification of effects that small molecules and patterning factors have on promoting OPC differentiation. In addition, the high capacity and simplicity of the hOPC purification method could aid in establishing a human OPC-based drug-discovery platform for performing high-throughput screens for myelination promoting compounds.

PDGFRα expressing OLLCs from the mouse CNS have been well characterized using scRNA-seq analysis^4,38^, but similar analysis using human cells has not been reported. To define the transcriptional diversity and heterogeneity of PDGFRα expressing human OLLCs, we performed scRNA-seq on hESC-derived reporter cells purified at various timepoints after the initiation of differentiation. We also analyzed PDGFRα expressing cells from an independent, non-reporter hiPSC line. Unbiased clustering of the cells identified distinct clusters of OPCs, astrocytes, oligodendrocytes, and pericytes. We also identified sub-populations of OPCs that were defined by their developmental stage and commitment to either the OL or astrocyte lineages. A sub-population of hOPCs with enriched cytokine response signaling was also uncovered.

A variety of studies indicate that OPCs and pre-OLs, but not mature OLs, express PDGFR*α*^1,4,12,57^. Thus, it was surprising that a significant number of our PDGFR*α*^+^/tdTomato^+^ cells showed a strong correlation with OLs and astrocytes and expressed numerous mature markers (Fig. 4 and Table 1). It should be noted, however, that the *PDGFRα* mRNA in our single-cell data are not well detected in the clusters representing mature cells (Fig. 4d). Possible explanations, given that the tdTomato and Thy1.2 proteins were obviously present at the time of purification, are that (1) PDGFR*α* protein is present but *PDGFRα* mRNA is not actively expressed in the mature cells at the time of purification, (2) the half-life of tdTomato^58^ and Thy1.2 is longer than PDGFR*α*^59^, allowing purification of cells that no longer express *PDGFRα* mRNA, or (3) perhaps, in the mature cells, *PDGFRα* mRNA is expressed as a low abundance transcript, and such transcripts are often missed in scRNA-seq^16,17,60^.

Although the DropSeq approach we used does not capture mature miRNAs because they are not poly-adenylated, DropSeq can sometimes capture pri-miRNAs. We identified a number of pre-miRNAs including pre-miR219-A2, which is highly enriched in human OLs^5,61^ and has previously been shown to be important for myelination and remyelination in mice^62–64^. Our PD-TT reporter system could thus be a useful resource for future studies to more directly explore ALC and OLLC specific miRNAs.

Additionally, the utility of our scRNA-seq data is further supported by IPA analysis, which revealed pathways associated with hOL and astrocyte differentiation. Of particular interest is the finding of enrichment of the CXCR4, Sphingosine-1-phosphate, integrin, mTOR, and cholesterol biosynthesis signaling pathways in mature OL cells. Pharmacologically modulating the cholesterol biosynthesis pathway (CBP) increased the amount of MBP mRNA and enhanced OL differentiation. Inhibition of CBP has been shown to cause accumulation of 8,9-unsaturated sterols^50,52^. Since these compounds increase the amount of MBP mRNA, it is possible that the 8,9-unsaturated sterols target upstream of MBP to increase its production. Transcriptomic study of the human OPCs treated with these compounds or supplemented with the 8,9-unsaturated sterols would help identify the upstream regulators of MBP expression. Moreover, further studies to modulate the other pathways identified in this study could potentially help in remyelination-based drug-discovery efforts.

The long-term expression of PDGFRα-tdTomato/Thy1.2 in the reporter OLLCs allowed us to perform pseudotime analysis and study their differentiation trajectories. The pseudotime analysis revealed OLs vs astrocytes as the two major lineage trajectories of the PDGFRα^+^ hOPCs. Oligodendrogenesis and astrogenesis from our PDGFRα-tdTomato^+^ cells are similar to in vitro cultured, PDGFRα^+^ primary hOPCs^11^, but contrasts with a recent report that suggests that Pdgfr*α*^+^ mouse OPCs can give rise to OLs, neurons or VLMC-pericytes but not astrocytes in vivo^38^. We did not find any study that discusses if astrocytes can arise from in vitro cultured Pdgfr*α*^+^ mouse OPCs. However, data suggesting that Sox10^+^ mouse OPCs can differentiate into astrocytes in vivo as well in vitro have been presented^65^. Our analysis, although keeping in mind that it is an in vitro study with hESC-derived *PDGFRα*^+^ and O4^+^ cells, and that the findings need to be confirmed in vivo, also suggests that astrocytes can originate from hOPCs.

We also identified transcription factors (TFs) that potentially help modulate lineage specification, differentiation, and maturation of OPCs into either OLs or astrocytes. Continued upregulation of the TFs *ZEB2, TSC22D4, ARID4B, PARP1, E2F3, SOX10, TCF7L2, TSC22D1, RBPJ, ARHGAP35, SOX4, SOX11, MLLT11, RBM22, ZNF711, EZH2,* and *DACH2*, which are enriched in OLs and downregulated in astrocyte cells, and *HES1, EGR1, FOSB, NFIA, NR2F1, ID3, KLF6*, and *ZFP36L1*, whose expression is enriched in astrocytes and decreased in OLs, seems to drive specification of OLs vs astrocytes from PDGFR*α*^+^ OPCs. The role of the majority of these TFs in OL/astrocyte differentiation and maturation has not been studied. Since the function of *SOX10* and *TCF7L2* in OL development and *NFIA* and *NR2F1* in astrocyte differentiation is well known^66,67^, and a crucial role of *ZFP36L1* in OL-astrocyte lineage transition was recently reported^68^, it is conceivable that the TFs we identified have important roles in OL vs astrocyte lineage specification. Loss of function and gain of function studies of these genes and TFs in OPCs will help to further validate their potential role in lineage specification of human OPCs and provide valuable information to our current understanding of OL and astrocyte differentiation.

Although mouse and human OPCs and OLs share transcriptomic similarity and conserved pathways, there appear to be some important species-specific distinctions with respect to their transcriptome, development, and regeneration^7^. In vivo, human gliogenesis starts during gestation and myelination can occur until age 22; while in rodents, gliogenesis occurs postnatally and myelination is resolved by day 60^69,70^. In vitro, *Ascl1* and FGF2 promote the generation of OLs from rodent OPCs but not from human OPCs^10^. In addition, unlike in animal models of MS where myelin is regenerated by newly formed oligodendrocytes, the capacity to generate oligodendrocytes around the lesions of human patients is generally diminished, and the limited remyelination that does occur at MS lesions is likely generated by pre-existing OLs^22^. Therefore, for improved disease modeling and to support drug-discovery efforts, it is crucial to expand our understanding of human OL maturation and better define the similarities and differences between murine and human OL differentiation. We hope that the hOPC purification system described in this manuscript along with our scRNA-seq data set (http://zacklab.org/OPCs/) will help provide the basis for ongoing and future studies that will more fully define the molecular mechanisms of human OL differentiation, maturation, and myelination.

## Methods

### Human pluripotent stem cells (PSCs) and culture conditions

hESC line WA09 (WiCell), an NIH-approved hESC line (NIH approval number: NIHhESC-10-0062 and WiCell agreement number: 09-W571), was used for this study. hESCs were maintained in StemFlex media (A3349401, ThermoFisher Scientific) on growth factor-reduced Matrigel (354230, Corning) coated plates at 37 °C, 10% CO_2_/5% O_2_. However, during reporter cell-line generation, the hESCs were maintained in mTeSR1 media (Stemcell Technologies). The iPSCs were always maintained in mTeSR1 media in normal culture conditions (37 °C, 5% CO_2_). hPSC colonies were passaged by dissociating with Accutase (A6964, Sigma-Aldrich). Cells were maintained in stem cell media containing 5 mM blebbistatin (B0560, Sigma-Aldrich) for the first 24 h after passaging, to improve single-cell survival.

Karyotype analysis was performed using a qPCR based hPSC Genetic Analysis Kit (StemCell Technologies, #07550). If significant aberration was detected, further analysis with G-banding (CellLine Genetics) was performed. Chromosomal aberration in hPSCs is common, with hESCs often having duplication in chromosome 1q and 20q as they get to higher passage number. We also observed isochromosome duplication of chromosome 1q as early as passage 10, ~3 months after generation of the line. Of note, the WA09 cells were already at p30 when purchased from WiCell. 10 out of 20 clones analyzed by G-banding had this abnormality. Cells were routinely tested for mycoplasma contamination (MycoAlert, Lonza) and only the cells free of contamination were used for OPC differentiation.

### Cloning

A guide sequence targeting the stop codon of the *PDGFRα* locus was designed in Deskgen.com. Guide sequence with minimal off-target and very high activity score was chosen and cloned into the BbsI restriction site of the Cas9 plasmid (Cas9-P2A-Puro modified from Addgene #62988^27^). pSpCas9(BB)-2A-Puro (PX459) V2.0 was a gift from Feng Zhang (Addgene plasmid # 62988; http://n2t.net/addgene:62988). To clone the donor plasmid, a ~2 kb PCR product was amplified from genomic DNA extracted from H9 ES cells and cloned into Zero Blunt TOPO cloning vector (ThermoFisher Scientific). The tdTomato-P2A-Thy1.2 reporter DNA sequence was then introduced into the TOPO-based donor plasmid, precisely upstream of the *PDGFRα* stop codon using Gibson assembly (New England Biolabs).

### Generation of PDGFRα reporter cell line

Gene editing and reporter cell line generation was performed using a transient antibiotic selection method^27^. Cells were transfected using the Lipofectamine Stem (STEM00001, ThermoFisher Scientific) transfection reagent following the manufacture’s recommended protocol. 0.35 μg Cas9 plasmid (Cas9-P2A-Puro modified from Addgene #62988) containing a gRNA sequence targeting the stop codon of the PDGFRα locus and 0.75 μg of donor plasmid were used for transfection. Roughly 40 h after transfection, the cells were selected with 0.6 μg/ml of puromycin for 24 h. Selected cells were passaged at 500–1000 single cells per well of a 6-well plate for colony formation followed by colony picking and PCR analysis^27^. PCR was performed using the Phusion Flash mastermix (ThermoFisher Scientific) and a 2-step PCR protocol following the manufacturer’s instruction. To generate the PDTT line, we picked 30 single-cell-derived colonies, of which, 7 had clear homozygous knock-In (Supplementary Fig. S1a). 5 of the homozygous colonies were further passaged and single-cell colonies picked again to confirm the homogeneous population of the knock-in cells (Supplementary Fig. S1b). Two clones from each of the 5 lines were then differentiated for 12 days to confirm tdTomato expression under a fluorescent microscope. Since all the clones seem to perform equally well, three clones were further expanded and stored as PDTT reporter cell lines. Of those, only one clone was used for differentiation and single-cell transcriptomic analysis.

For off-target analysis, the top 5 potential off-target locations were chosen based on Deskgen.com, the website used to design the guide sequence. Specific primers upstream and downstream of the location were designed (Supplementary Table 1). PCR followed by Sanger sequencing was performed to confirm no off-target mutation was caused during genome editing.

### Oligodendrocyte differentiation protocol

hESCs and hiPSCs were differentiated into OPCs and OLs as previously described^30^ with minor modifications. Briefly, hESCs were dissociated to single cells and plated on Matrigel-coated plate at 100,000 cells/well of a 6-well plate and maintained in StemFlex media at 37 °C, 10% CO_2_/ 5% O_2_ (hiPSCs were maintained in mTeSR1 media at 37 °C, 5% CO_2_). Two days after passaging, neural differentiation and spinal cord patterning were induced through dual SMAD inhibition and the addition of 100 nM all-trans RA^71^. From days 8 to 12, differentiating cells were maintained in neural induction media supplemented with RA (100 nM) and SAG (1 mM). On day 12, adherent cells were lifted and cultured in low-attachment plates to favor sphere aggregation. On day 30, spheres were plated into poly-L-ornithine/laminin-coated dishes in a media supplemented with, B27 (ThermoFisher, 12587010), N2 supplement (ThermoFisher, 17502048), PDGF-AA (221-AA-10, R&D systems), neurotrophin-3, HGF (294-HG-025 R&D systems), and T3. Detailed information on each reagent used is listed in supplementary Table 2. Once a significant number of tdTomato^+^ OPCs were visible around days 65–70, a mitogen-free glial medium was used to drive oligodendrocyte maturation.

### Cryopreservation and revival of hPSCs and purified OPCs

For cryopreservation, hPSCs were dissociated with accutase, diluted in DMEM/F12, and centrifuged at 150 × *g* for 5 min. Cell pellets were resuspended at 1 million cells/ml in CryoStor CS10 (07930, Stemcell Technologies), placed in cryovials, and stored at −80 °C. MACS purified hOPCs were pelleted by centrifuging at 250 × *g* for 5 min and cryopreserved in the CryoStor at 5 million/ml. hPSCs were revived by incubating cryopreserved cells at 37 °C for 3 min, washing with 10 ml of DMEM/F12, resuspending in mTesr or StemFlex medium supplemented with blebbistatin, and plating them in Matrigel-coated plate. For cryopreserved purified hOPCs, cells were thawed at 37 °C for 3 min, resuspended in of PDGF media, and directly plated on PLO-laminin-coated dish without centrifuging/washing the cells. The cells were then allowed to settle and attach for 2 h. After 2 h, media was changed very gently to remove DMSO, which is part of CryoStor buffer. We noticed that washing the cryopreserved cells can lead to ~40% loss of cells, which fail to precipitate and form pellets. With the direct plating method described here, the purified and cryopreserved OPCs can be revived with >80% retention and survival. However, we notice a significant loss of cells if passaged after purification or after revival from cryopreservation, therefore, we do not recommend passaging the hOPCs after purification or revival.

### Flow cytometry and MACS purification of the reporter hOPCs

Flow cytometry analysis and MACS purification were performed as previously described^24^ with the following modifications. Cells were dissociated into a single-cell suspension by incubating in accutase for ~45 min. The single-cell suspension was then passed through a ~70 uM cell strainer (BD Biosciences), washed, and resuspended in Live Cell Imaging Solution (ThermoFisher Scientific) for analysis with an SH800S Cell Sorter (Sony Biotechnology, San Jose, CA). For flow analysis, BSC and FSC were used to select and subset live cells, and only live cells were used to quantify a number of tdTomato^+^ cells. A gate was set up using WT hES cells differentiated to day 95. Since it is not practical to differentiate a WT line every time a reporter line is differentiated, the same gate was used for every flow analysis. For MACS purification, cells were resuspended in MACS buffer after passing through the cell strainer. A CD90.2 (THY1.2), O4, or A2B5 MicroBeads were added to the cell suspension and incubated at room temperature for 15 min for cell binding. Cells were generally run through the MS column twice without additional supplementation of MicroBeads to increase the purity and achieve ~90% tdTomato^+^ cells. All MACS reagents were purchased from Miltenyi Biotec (Auburn, CA) and manufacturer instructions were followed.

### Testing pharmacological compounds on hOPCs

Day 85 hOPCs were MACS purified to ~90% purity. 200,000 purified hOPCs were plated per well of a PLO/laminin-coated 24 well tissue culture plate, in a mitogen-free glial media. Day after plating the cells, culture media was replaced with media containing different compounds or DMSO. Ketoconazole, amorolfine, and tasin-1, which target CYP51A1, TM7SF2, and EBP, respectively, were used to target the cholesterol biosynthesis pathways; CYM5520 was used to target S1P; WZ811 was used to inhibit CXCR4 signaling, and rapamycin was used to inhibit mTOR signaling. Based on previous publications and our lab’s unpublished screening work, following optimal dose for each compound was chosen: (a) CYM5520 (1.2 μM) (b) WZ811 (1 μM) (c) Ketoconazole (370 nM) (d) Amorolfine (370 nM) (e) Tasin-1 (41 nM) (f) Rapamycin (123 nM). All the compounds were purchased from Selleckchem (selleckchem.com). Media containing the compounds was replaced on day 3, and on day 7 the cells were either lysed for RNA extraction and qRT-PCR or fixed with 4% paraformaldehyde for immunostaining.

### Immunofluorescence staining, microscopy, and qRT-PCR

All sequences for qRT-PCR primers can be found in Supplementary Table S1. Total RNA was isolated using the RNeasy Mini Kit (QIAGEN) and reverse transcribed using the High-Capacity cDNA Reverse Transcription Kit (Applied Biosystems). A 2uL PCR reaction was set up using an acoustic liquid handler (ECHO 550, Labcyte) and performed with the CFX384 real-time PCR instrument (Bio-Rad). Assays included at least two technical and two biologicals replicates and were run using the Sso Advanced Universal SYBR Green Supermix (Bio-Rad). Primers used for the qRT-PCR are listed in Supplementary Table 1.

For immunofluorescence staining, cells were fixed with 4% paraformaldehyde, and simultaneously permeabilized and blocked with 0.2% Triton X-100 + 5% BSA + 5% normal goat serum (or serum specific to the host of secondary antibody) for an hour. Cells were then incubated with the appropriate dilution of primary antibodies over-night followed by secondary antibodies for 2 h. Antibodies used for the study are listed in Supplementary Table 2. Fluorescence images were taken using either the EVOS FL Auto 2 (ThermoFisher Scientific) or Zeiss 510 confocal microscope. Co-staining analysis of the tdTomato and PDGFRa was performed using a built-in algorithm of the ArrayScan image analysis software (ThermoFisher Scientific ArrayScan XTI). Thirteen images were used for analysis.

### Live cell imaging

The EVOS FL Auto 2 Cell Imaging System (ThermoFisher Scientific) was used for imaging cells in culture over time for time-lapse videos. Cells were maintained in a live cell chamber at 37 °C with 5% CO_2_ and 85% humidity and areas around neurospheres were scanned every 20 min. Images were compiled at 15 fps using ImageJ to generate time-lapse videos.

### Statistical analysis

All qRT-PCR data are presented as fold change in RNA normalized to the expression of two housekeeping genes: either *GAPDH* and *SRT72* or *GAPDH* and *CREBBP*. qRT-PCR data were analyzed using CFX Maestro (Bio-Rad) qPCR analysis software and graphed using Prism (GraphPad, V9).

### Drop-seq-based single-cell capture and RNA-sequencing

Drop-seq-based single-cell RNA-seq was performed as previously described by Macosko et al.^16^. Barcoded microparticles were purchased from Chemgenes Corporations. During differentiation, a large number of differentiating cultures are PDGFRa^+^ on day 75, providing sufficient cell numbers for downstream analysis^14,30^. We observe a similar phenomenon with tdTomato-expressing cells in our differentiation culture. Therefore, day 75 and two other timepoints, two weeks apart were considered for time-course single-cell capture. Since MACS purification and Drop-seq could not be timed on the exact intended day, we ended up using cells from days 77, 89, and 104. The differentiating cells were MACS purified for Thy1.2 expression. The days 89 and 104 are from a single batch of differentiation and d77 and O4^+^ cells are from two different batches of differentiation. Small fraction of the purified cells was used for FACS-based analysis to confirm that >90% of them were tdTomato^+^. Cells were MACS purified 2–3 times until ~90% purity was achieved. Using the microfluidic device, the purified reporter cells were captured into ~1 nl size droplets containing barcoded nanoparticles and lysis buffer. Generated droplets were broken with perfluorooctanol (Sigma, 370533) in 30 ml of 6× SSC. The beads were then washed, reverse transcribed, PCR amplified, and the amplified cDNA quantified using a BioAnalyzer High Sensitivity Chip (Agilent). The cDNA was then fragmented and amplified for 3′ prime end sequencing with the Nextera XT DNA sample prep kit (Illumina). cDNA and libraries for each sample were prepared independently, and an equimolar amount of each library was then pooled together for sequencing. The libraries were purified, quantified, and then sequenced on the rapid flow chip in Illumina HiSeq 2500. From the three independent timepoints, a combined total of ~4800 purified cells were captured.

### Bioinformatic analysis

#### Quality control and clustering and differential gene expression

The principal component analysis (PCA) and *t*-distributed stochastic neighbor embedding (tSNE) analyses were performed using a previously published R package, Seurat^72^. As a quality control, only cells that had a minimum of 250 mRNA molecules and a maximum of 20% mitochondrial RNA were used for analysis. In order to eliminate probable doublets from the data set, we bioinformatically filtered out cells with >30,000 unique molecular identifier (UMIs) (Supplementary Fig. S3a). Additionally, we removed cells that exhibited (1) expression of <250 genes or (2) >20% mitochondrial gene content^72^. The remaining 3271 cells were used for further analysis. Genes that were expressed in a minimum of 3 cells were included for the analysis. 1874 highly variable genes were input for PCA analysis, and the 16 statistically significant PC’s were used for clustering and UMAP or *t*-SNE embedding. To identify differentially expressed genes for each cluster, genes that are expressed in less than 10% in either group or have a log fold change of <0.25 are filtered. For the rest of the genes, Wilcoxon rank-sum tests are performed with multiple comparison adjustments. All 1266 enriched genes were used to generate the expression heatmap (Fig. 4c), and the top 40 most significant genes for each OPC sub-cluster with adjusted *P*-value below 0.05 are used for the OPC-restricted heatmap (Fig. 5c).

#### Data integration and label transfer

Annotated UMI count matrices from the control samples of adult and fetal human brain were retrieved from the GEO (GSE118257, GSE104276) and Bioproject (544731)^32–34^ and processed independently. Neurons, phagocytes, microglia were excluded from the analysis and remaining cell-types from all three data set were integrated using Seurat Anchor (3.0)^73^ with CCA dimension reduction and using 2000 variable features for anchor identification. During the integration, the fetal data set is first merged with one of the adult data sets, the combined data set was then merged with another adult data set. For the integrated data set, k-Nearest Neighbor graphs are constructed using top 30 PCs, and 14 clusters are identified using Leiden modularity optimization with a resolution of 0.8. In the integrated data set, OPCs and astrocytes from the three data sets^73^ merged with each other. Oligodendrocytes from the adult samples merged in the 2-dimensional UMAP embedding, but emerged as relatively distinct clusters in Leiden clustering. Neuro Progenitors and Pericyte/Endothelial cells formed their own clusters as there are no counterparts in the other data sets. Each of the 14 clusters are then given a new cluster label, with 7 Oligodendrocyte clusters, 3 Astrocyte clusters, 2 OPC clusters, 1 Neuro Progenitor cluster and 1 Pericyte/Endothelial cluster being assigned. Next, we carried out integration and label transfer of cells in our data set with the reference using Seurat Anchor (3.0) with CCA dimension reduction using a total of 1568 genes that are variable in the reference and also detected in our data. The cluster assignment probabilities are then transferred for each cell in our data set by weighting labels of 20 closest neighbors in the CCA space (Supplementary Fig. S4), and for each cluster in our data set, an average of the label assignment probability is calculated (Fig. 4e, f)

To generate a co-embedding of tdTom and the reference (Fig. 4b), normalized gene expression values of the variable genes are also transferred for each cell in tdTom data similar to the labels. The resulting data are then combined with the reference gene expression. Gene-wise scaling, PCA, and UMAP are subsequently performed on the combined data.

#### G2M score calculation and gene set enrichment analysis

Cell cycle scoring was performed with a list of cell cycle genes collected from Tirosh et al. (Supplementary Table S5). The cycling progenitor clusters are cycling and the other clusters were non-cycling. CyP1 was G2M phase while CyP2 and CyP3 are in S phase. To conduct GSEA analysis on the differentially expressed genes, enriched genes are first ranked by the product of −log10(*P*-value) and log (average fold change). The gene ranks are used for pre-ranked GSEA analysis using all Gene Ontology terms, with 1000 permutations^74^.

#### Pseudotemporal trajectory analysis

Time-series analysis to generate a pseudotemporal trajectory was performed using an unsupervised differential gene expression test based on sample age in Monocle, following previously published detailed instructions^19,75^. The top 752 genes differentially expressed based on age were used for ordering and trajectory reconstruction. Differential gene expression was performed on each node of the resulting trajectory to identify genes with branch-dependent expression. Differential gene expression was performed using either Seurat or Monocle.

#### Calculation of Spearman correlations and human-mouse overlap assessment

Total expression of all the genes expressed in both our data set and the previously published snRNAseq data set^32^ (13,658 total genes) was used to normalize gene expression in each cell. The normalized expression was averaged across each population. 1195 variable genes expressed in both data sets were then used to calculate the Spearman correlation between each population. To combine all the oligodendrocyte sub-population as one population, expression was weighted by the number of cells in each sub-population. A similar weighted average was also used to combine the two astrocyte sub-populations. For the comparison with bulk RNA-seq data set, 767 highly variable genes from each human tissue-type published by Zhang et al.^44^ were compared to each of our population’s averaged normalized expression. For comparison with mouse cells, all the genes expressed in each of our clusters were compared to the genes from each of the mouse CNS cell-type previously published^3^.

To assess the overlap of enriched genes in our hESC-derived OLLCs, ALCs, OPCs, and endothelial/pericyte cells to that of the enriched genes in the corresponding population from the mouse data set, all 1266 differentially expressed genes used for the expression heatmap (Fig. 4b) were used for the comparison. From the mouse data set^3^, genes expressed at levels >20 FPKM and >5-fold relative to the average expression in all other populations, were considered enriched.

Codes for the bioinformatic analysis of the single-cell data are deposited in GitHub^76^.

#### The networks and pathway analyses

The networks and pathway analyses were generated through the use of Ingenuity Pathway Analysis (IPA QIAGEN Inc)^77^. For the analysis, differentially expressed genes and their corresponding *P*-value and fold change (from Supplementary Table 1) were uploaded for each cluster. The sterol biosynthesis pathway map was generated using KEGG via Visualization and Integrated Discovery (DAVID) tools and its steroid biosynthesis pathway as the reference pathway.

### Reporting summary

Further information on research design is available in the Nature Research Reporting Summary linked to this article.

## Supplementary information

Supplementary Information

Reporting Summary

Description of Additional Supplementary Files

Supplementary Movie 1

Supplementary Movie 2

Supplementary Data 1

Supplementary Data 2

Supplementary Data 3

Supplementary Data 4

Supplementary Data 5

Supplementary Data 6

Supplementary Data 7

## Data Availability

RNA-Seq data generated for this paper are deposited to the NCBI’s Gene Expression Omnibus (GEO) database with accession number GSE146373. Following publicly available data sets (GSE118257, GSE104276, GSE52564, and GSE73721) and Bioproject (544731) were also used for this study. Figures associated with the RNA-seq data are Figs. 4–8; and Supplementary Figs. S4–S12. List of genes, GSEA, and IPA for each scRNA-seq-based cluster are available as Supplementary Data 1–7. GSEA and gene expression data are also available for viewing at http://zacklab.org/OPCs/. Source data are provided with this paper.

## Source data

Source Data

## Code availability

The codes used to generate the results reported in this manuscript can be accessed at https://github.com/wefang/TdTom^76^. Previously unreported custom codes were not used in the manuscript.

## References

[1] Baumann N, Pham-Dinh D (2001). Biology of oligodendrocyte and myelin in the mammalian central nervous system. Physiol. Rev..

[2] Duncan ID, Radcliff AB (2016). Inherited and acquired disorders of myelin: the underlying myelin pathology. Exp. Neurol..

[3] Zhang Y (2014). An RNA-sequencing transcriptome and splicing database of glia, neurons, and vascular cells of the cerebral cortex. J. Neurosci..

[4] Marques S (2016). Oligodendrocyte heterogeneity in the mouse juvenile and adult central nervous system. Science.

[5] de Faria O (2012). Regulation of miRNA 219 and miRNA Clusters 338 and 17-92 in Oligodendrocytes. Front Genet.

[6] Elbaz, B. & Popko, B. Molecular control of oligodendrocyte development. *Trends Neurosci.*10.1016/j.tins.2019.01.002 (2019). 10.1016/j.tins.2019.01.002 PMC7397568 30770136

[7] Dietz KC, Polanco JJ, Pol SU, Sim FJ (2016). Targeting human oligodendrocyte progenitors for myelin repair. Exp. Neurol..

[8] Chanoumidou, K., Mozafari, S., Baron-Van Evercooren, A. & Kuhlmann, T. Stem cell derived oligodendrocytes to study myelin diseases. *Glia*10.1002/glia.23733 (2019). 10.1002/glia.23733 31633852

[9] Sim FJ, Windrem MS, Goldman SA (2009). Fate determination of adult human glial progenitor cells. Neuron Glia Biol..

[10] Wang J (2014). Transcription factor induction of human oligodendrocyte progenitor fate and differentiation. Proc. Natl Acad. Sci. USA.

[11] Sim FJ (2011). CD140a identifies a population of highly myelinogenic, migration-competent and efficiently engrafting human oligodendrocyte progenitor cells. Nat. Biotechnol..

[12] Goldman SA, Kuypers NJ (2015). How to make an oligodendrocyte. Development.

[13] Nistor GI, Totoiu MO, Haque N, Carpenter MK, Keirstead HS (2005). Human embryonic stem cells differentiate into oligodendrocytes in high purity and myelinate after spinal cord transplantation. Glia.

[14] Douvaras P (2014). Efficient generation of myelinating oligodendrocytes from primary progressive multiple sclerosis patients by induced pluripotent stem cells. Stem Cell Rep..

[15] Marton RM (2019). Differentiation and maturation of oligodendrocytes in human three-dimensional neural cultures. Nat. Neurosci..

[16] Macosko EZ (2015). Highly parallel genome-wide expression profiling of individual. Cells Using Nanoliter Droplets. Cell.

[17] Zheng GX (2017). Massively parallel digital transcriptional profiling of single cells. Nat. Commun..

[18] van Bruggen D, Agirre E, Castelo-Branco G (2017). Single-cell transcriptomic analysis of oligodendrocyte lineage cells. Curr. Opin. Neurobiol..

[19] Trapnell C (2014). The dynamics and regulators of cell fate decisions are revealed by pseudotemporal ordering of single cells. Nat. Biotechnol..

[20] Wang S (2013). Human iPSC-derived oligodendrocyte progenitor cells can myelinate and rescue a mouse model of congenital hypomyelination. Cell Stem Cell.

[21] Piao J (2015). Human embryonic stem cell-derived oligodendrocyte progenitors remyelinate the brain and rescue behavioral deficits following radiation. Cell Stem Cell.

[22] Yeung, M. S. Y. et al. Dynamics of oligodendrocyte generation in multiple sclerosis. *Nature*10.1038/s41586-018-0842-3 (2019). 10.1038/s41586-018-0842-3 PMC6420067 30675058

[23] Traiffort, E., Zakaria, M., Laouarem, Y. & Ferent, J. Hedgehog: a key signaling in the development of the oligodendrocyte lineage. *J. Dev. Biol.* **4**, 10.3390/jdb4030028 (2016). 10.3390/jdb4030028 PMC5831774 29615592

[24] Sluch VM (2017). Enhanced stem cell differentiation and immunopurification of genome engineered human retinal ganglion cells. Stem Cells Transl. Med..

[25] Wang Y, Wang F, Wang R, Zhao P, Xia Q (2015). 2A self-cleaving peptide-based multi-gene expression system in the silkworm Bombyx mori. Sci. Rep..

[26] Daniels RW, Rossano AJ, Macleod GT, Ganetzky B (2014). Expression of multiple transgenes from a single construct using viral 2A peptides in Drosophila. PLoS ONE.

[27] Sluch VM (2018). Highly efficient scarless knock-in of reporter genes into human and mouse pluripotent stem cells via transient antibiotic selection. PLoS ONE.

[28] Devalle S (2012). Implications of aneuploidy for stem cell biology and brain therapeutics. Front. Cell Neurosci..

[29] Rebuzzini P, Zuccotti M, Redi CA, Garagna S (2015). Chromosomal abnormalities in embryonic and somatic stem cells. Cytogenet. Genome Res..

[30] Douvaras P, Fossati V (2015). Generation and isolation of oligodendrocyte progenitor cells from human pluripotent stem cells. Nat. Protoc..

[31] Byrne SM, Ortiz L, Mali P, Aach J, Church GM (2015). Multi-kilobase homozygous targeted gene replacement in human induced pluripotent stem cells. Nucleic Acids Res..

[32] Jakel S (2019). Altered human oligodendrocyte heterogeneity in multiple sclerosis. Nature.

[33] Schirmer L (2019). Neuronal vulnerability and multilineage diversity in multiple sclerosis. Nature.

[34] Zhong S (2018). A single-cell RNA-seq survey of the developmental landscape of the human prefrontal cortex. Nature.

[35] Velasco S (2019). Individual brain organoids reproducibly form cell diversity of the human cerebral cortex. Nature.

[36] Fujiwara K, Jindatip D, Kikuchi M, Yashiro T (2010). In situ hybridization reveals that type I and III collagens are produced by pericytes in the anterior pituitary gland of rats. Cell Tissue Res..

[37] Seet LF (2017). Upregulation of distinct collagen transcripts in post-surgery scar tissue: a study of conjunctival fibrosis. Dis. Model Mech..

[38] Marques S (2018). Transcriptional convergence of oligodendrocyte lineage progenitors during development. Dev. Cell.

[39] Kirby L (2019). Oligodendrocyte precursor cells present antigen and are cytotoxic targets in inflammatory demyelination. Nat. Commun..

[40] Falcao AM (2018). Disease-specific oligodendrocyte lineage cells arise in multiple sclerosis. Nat. Med..

[41] Fernandez-Castaneda A (2020). The active contribution of OPCs to neuroinflammation is mediated by LRP1. Acta Neuropathol..

[42] Morales Pantoja IE (2020). iPSCs from people with MS can differentiate into oligodendrocytes in a homeostatic but not an inflammatory milieu. PLoS ONE.

[43] Starost L (2020). Extrinsic immune cell-derived, but not intrinsic oligodendroglial factors contribute to oligodendroglial differentiation block in multiple sclerosis. Acta Neuropathol..

[44] Zhang Y (2016). Purification and characterization of progenitor and mature human astrocytes reveals transcriptional and functional differences with mouse. Neuron.

[45] Lee HK (2015). Apcdd1 stimulates oligodendrocyte differentiation after white matter injury. Glia.

[46] Kelley KW, Nakao-Inoue H, Molofsky AV, Oldham MC (2018). Variation among intact tissue samples reveals the core transcriptional features of human CNS cell classes. Nat. Neurosci..

[47] Carbajal KS, Miranda JL, Tsukamoto MR, Lane TE (2011). CXCR4 signaling regulates remyelination by endogenous oligodendrocyte progenitor cells in a viral model of demyelination. Glia.

[48] Coelho RP, Saini HS, Sato-Bigbee C (2010). Sphingosine-1-phosphate and oligodendrocytes: from cell development to the treatment of multiple sclerosis. Prostaglandins Other Lipid Mediat..

[49] O’Meara RW, Michalski JP, Kothary R (2011). Integrin signaling in oligodendrocytes and its importance in CNS myelination. J. Signal Transduct..

[50] Hubler Z (2018). Accumulation of 8,9-unsaturated sterols drives oligodendrocyte formation and remyelination. Nature.

[51] Etienne-Manneville S, Hall A (2001). Integrin-mediated activation of Cdc42 controls cell polarity in migrating astrocytes through PKCzeta. Cell.

[52] Wahl SE, McLane LE, Bercury KK, Macklin WB, Wood TL (2014). Mammalian target of rapamycin promotes oligodendrocyte differentiation, initiation and extent of CNS myelination. J. Neurosci..

[53] Bento-Abreu A (2009). Albumin endocytosis via megalin in astrocytes is caveola- and Dab-1 dependent and is required for the synthesis of the neurotrophic factor oleic acid. J. Neurochem..

[54] Edmond J, Robbins RA, Bergstrom JD, Cole RA, de Vellis J (1987). Capacity for substrate utilization in oxidative metabolism by neurons, astrocytes, and oligodendrocytes from developing brain in primary culture. J. Neurosci. Res..

[55] Bullen, C. K. et al. Infectability of human BrainSphere neurons suggests neurotropism of SARS-CoV-2. *ALTEX*10.14573/altex.2006111 (2020). 10.14573/altex.2006111 32591839

[56] Wang, J. et al. Robust myelination of regenerated axons induced by combined manipulations of GPR17 and microglia. *Neuron*10.1016/j.neuron.2020.09.016 (2020). 10.1016/j.neuron.2020.09.016 PMC7736523 33108748

[57] Ellison JA, de Vellis J (1994). Platelet-derived growth factor receptor is expressed by cells in the early oligodendrocyte lineage. J. Neurosci. Res..

[58] Muzumdar MD, Tasic B, Miyamichi K, Li L, Luo L (2007). A global double-fluorescent Cre reporter mouse. Genesis.

[59] Ho AL (2012). PDGF receptor alpha is an alternative mediator of rapamycin-induced Akt activation: implications for combination targeted therapy of synovial sarcoma. Cancer Res..

[60] Klein AM (2015). Droplet barcoding for single-cell transcriptomics applied to embryonic stem cells. Cell.

[61] Letzen BS (2010). MicroRNA expression profiling of oligodendrocyte differentiation from human embryonic stem cells. PLoS ONE.

[62] Wang H (2017). miR-219 cooperates with miR-338 in myelination and promotes myelin repair in the CNS. Dev. Cell.

[63] Dugas JC (2010). Dicer1 and miR-219 Are required for normal oligodendrocyte differentiation and myelination. Neuron.

[64] Zhao X (2010). MicroRNA-mediated control of oligodendrocyte differentiation. Neuron.

[65] Suzuki N (2017). Differentiation of oligodendrocyte precursor cells from Sox10-venus mice to oligodendrocytes and astrocytes. Sci. Rep..

[66] Zhao C (2016). Dual regulatory switch through interactions of Tcf7l2/Tcf4 with stage-specific partners propels oligodendroglial maturation. Nat. Commun..

[67] Ehrlich M (2017). Rapid and efficient generation of oligodendrocytes from human induced pluripotent stem cells using transcription factors. Proc. Natl Acad. Sci. USA.

[68] Weng Q (2019). Single-Cell transcriptomics uncovers glial progenitor diversity and cell fate determinants during development and gliomagenesis. Cell Stem Cell.

[69] Craig A (2003). Quantitative analysis of perinatal rodent oligodendrocyte lineage progression and its correlation with human. Exp. Neurol..

[70] Semple BD, Blomgren K, Gimlin K, Ferriero DM, Noble-Haeusslein LJ (2013). Brain development in rodents and humans: Identifying benchmarks of maturation and vulnerability to injury across species. Prog. Neurobiol..

[71] Chambers SM (2009). Highly efficient neural conversion of human ES and iPS cells by dual inhibition of SMAD signaling. Nat. Biotechnol..

[72] Satija R, Farrell JA, Gennert D, Schier AF, Regev A (2015). Spatial reconstruction of single-cell gene expression data. Nat. Biotechnol..

[73] Stuart T (2019). Comprehensive integration of single-cell. Data Cell.

[74] Subramanian A, Kuehn H, Gould J, Tamayo P, Mesirov JP (2007). GSEA-P: a desktop application for gene set enrichment analysis. Bioinformatics.

[75] Trapnell, C. *Monocole: Differential Expression and Time-series Analysis for Single-cell RNA-Se*, http://cole-trapnell-lab.github.io/monocle-release/articles/v2.2.0/ (2016).

[76] Fang, W., Xitiz, C., Kallman, A., Ji, H. & Zack, D. Single-cell transcriptomic analysis reveals molecular diversity and developmental heterogeneity of human stem cell-derived oligodendrocyte lineage cells. *Zenodo*10.5281/zenodo.4290971 (2020). 10.1038/s41467-021-20892-3 PMC7844020 33510160

[77] Kramer A, Green J, Pollard J, Tugendreich S (2014). Causal analysis approaches in ingenuity pathway analysis. Bioinformatics.

