# Supplementary material for: Single-cell transcriptomic reveals molecular diversity and developmental heterogeneity of human stem cell-derived oligodendrocyte lineage cells
Source: Nat Commun. 2021 Jan 28;12:652. doi: 10.1038/s41467-021-20892-3 (PMC7844020; doi:10.1038/s41467-021-20892-3)
Supplement: Supplementary file 1 — Supplementary Information [file 41467_2021_20892_MOESM1_ESM.pdf]

SUPPLEMENTARY INFORMATION- *Chamling et al.*

Single-Cell Transcriptomic Reveals Molecular Diversity and Developmental Heterogeneity of Human Stem Cell-derived Oligodendrocyte Lineage Cells

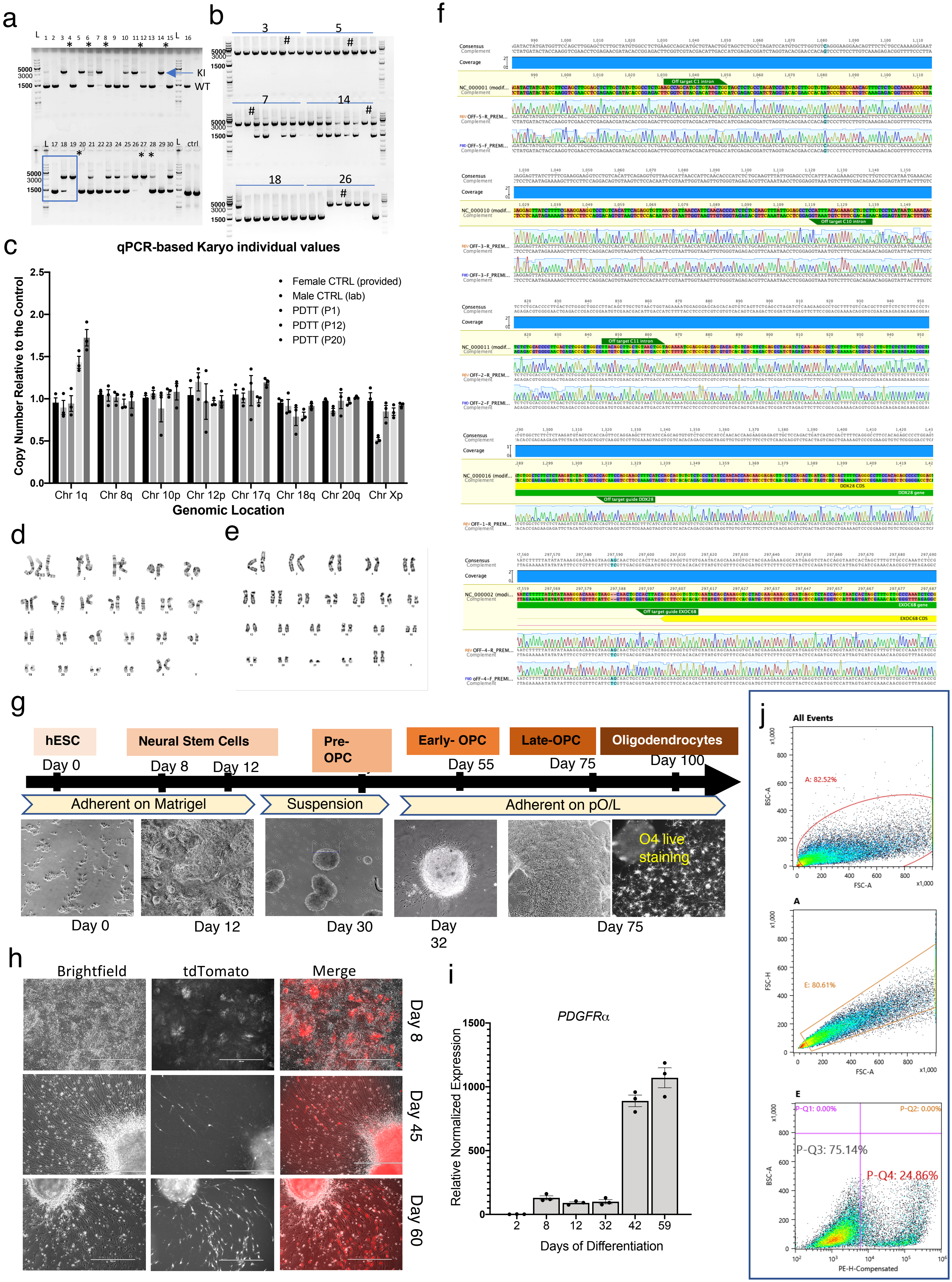
a
KI
WT
b
c
qPCR-based Karyo individual values
Female CTRL (provided)
Male CTRL (lab)
PDTT (P1)
PDTT (P12)
PDTT (P20)
Copy Number Relative to the Control
Chr 1q
Chr 8q
Chr 10p
Chr 12p
Chr 17q
Chr 18q
Chr 20q
Chr Xp
Genomic Location
d
e
f
Off target C1 intron
Off target C10 intron
Off target C11 intron
DDX28 CDS
DDX28 gene
Off target guide DDX28
Off target guide EXOC68
EXOC68 gene
EXOC68 CDS
g
hESC
Neural Stem Cells
Pre-OPC
Early- OPC
Late-OPC
Oligodendrocytes
Day 0
Day 8
Day 12
Day 55
Day 75
Day 100
Adherent on Matrigel
Suspension
Adherent on pO/L
O4 live staining
Day 0
Day 12
Day 30
Day 32
Day 75
h
Brightfield
tdTomato
Merge
Day 8
Day 45
Day 60
i
PDGFRα
Relative Normalized Expression
Days of Differentiation
j
All Events
A: 82.52%
BSC-A
FSC-A
A
E: 80.61%
FSC-H
E
P-Q1: 0.00%
P-Q2: 0.00%
P-Q3: 75.14%
P-Q4: 24.86%
PE-H-Compensated

Supplementary Figure 1. Generation and differentiation of the PDGFR∝ reporter (PD-TT) cell line. **a)** PCR-based genotyping of thirty single cell-derived colonies. Eighteen colonies seem to have at least some cells with the reporter sequence knocked-in, and at least eight of them appear to have homozygous integration (marked with *). Box represents the image that is included in Fig. 1b. **b)** Eight sub-colonies from six initial colonies were re-genotyped to ensure clonal homozygous knock-in. **c)** qPCR-based genetic analysis shows normal karyotype of the PDTT reporter cells at passage 1, but abnormality in the chromosome 1q after passage 12. Data is presented as mean ± SEM. This experiment was repeated once (n=1 biological sample, with 3 technical replicates) as a part of routine karyotype screen before sending samples for G-banding karyotyping. **d)** Further analysis by G-banding showed isochromosome duplication of long arm of chromosome 1 in 50% of the clones analyzed in cells as early as passage #10. **e**) Karyotype of the hiPSC line used for OPC differentiation and scRNAseq was normal. **f)** Top five potential off target locations were selected based on the prediction generated by Deskgen.com. The genomic regions were amplified by PCR and Sanger sequenced to show that there were no off-target mutations at these loci in the reporter line. Note that the discrepancy in the last sequence is due to a known polymorphism (Rs1553417099), an intron variant caused due to insertion of AG at chr2:72465144. **g)** Timeline of the differentiation protocol with images at different stages of differentiation. At Day 75, the majority of the OPCs that migrate out from the neurospheres plated on day 30 are O4 positive. **h)** tdTomato expression (driven by PDGFR∝) is observed as early as day 8 on the neuroepithelial layer of the differentiating culture. However, individual, /PDGFRα-tdTomato positive cells emerge from the neurospheres around day 45, rapidly divide and migrate out as seen in the day 60 culture. Scale bar: 400 μm. **g-h)** were independently repeated 10 times with similar results. **i)** qPCR analysis of the *PDGFRα* mRNA level coincides with the tdTomato expression in the cultures. n=3 independent differentiations, data is presented as mean ± SEM. **j)** An example of gating strategy used for the flow analysis of the tdTomato expressing cells.

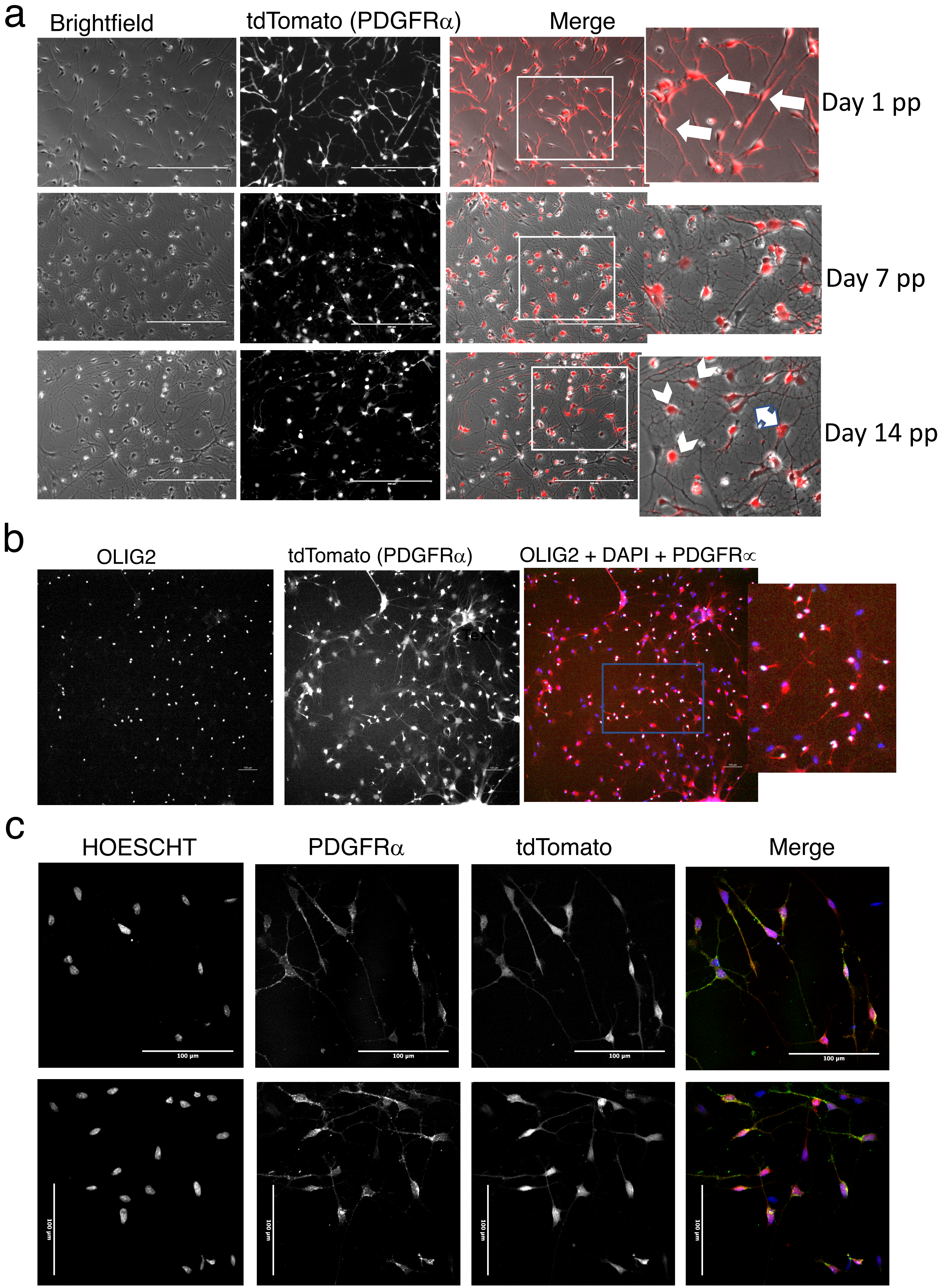

a
Brightfield
tdTomato (PDGFRα)
Merge
Day 1 pp
Day 7 pp
Day 14 pp
b
OLIG2
tdTomato (PDGFRα)
OLIG2 + DAPI + PDGFRα
c
HOESCHT
PDGFRα
tdTomato
Merge
100 µm

Supplementary Figure 2. Purified reporter cells express OPC markers and have the characteristics of PDGFRα+ OPC morphology. **a)** Day 80 MACS purified reporter cells plated on laminin coated surface and imaged 1-day post purification (pp) (**top**) are tdTomato+ and morphologically resemble bipolar OPCs (arrow). When cultured in mitogen free media, within 14 days, the cells start maturing (**bottom**). tdTomato expression is decreased and, in many cells, processes resembling OLs start emerging (arrowheads). Instead of thin processes, some cells are flatter with thicker branches resembling astrocytes (double arrow). pp: post purification. Scale bar 200 μm. **b)** Immunostaining to show the MACS purified tdTomato+ cells express OLIG2. Scale bar: 100 μm. **c)** High resolution (40X) images showing PDGFRα localizes to the cell membrane and tdTomato in the cytoplasm. Scale bar: 100 μm **a, b,** and **c** were independently repeated 5, 2 and 3 times respectively with similar results.

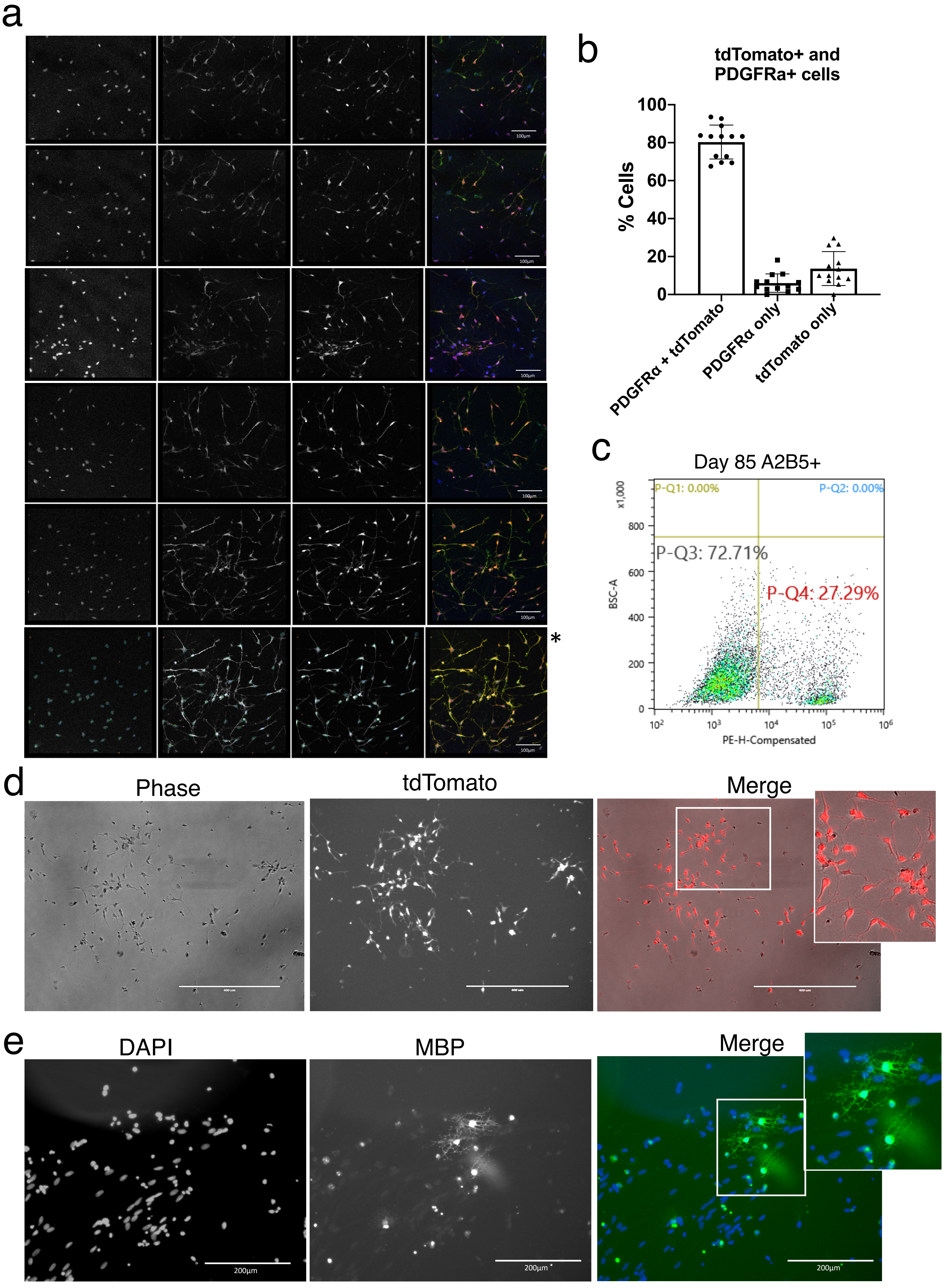

a
100µm
*
b
tdTomato+ and PDGFRa+ cells
% Cells
100
80
60
40
20
0
PDGFRα + tdTomato
PDGFRα only
tdTomato only
c
Day 85 A2B5+
x1,000
P-Q1: 0.00%
P-Q2: 0.00%
P-Q3: 72.71%
P-Q4: 27.29%
BSC-A
800
600
400
200
0
10^2
10^3
10^4
10^5
10^6
PE-H-Compensated
d
Phase
tdTomato
Merge
e
DAPI
MBP
Merge
200µm

Supplementary Figure 3. tdTomato+ OPCs express PDGFRα and maintain competence after freezing. a-b) Quantification of tdTomato and PDGFRα double positive cells. **a)** Example of the images used for quantification. The last panel marked with * shows example of masks that were used to quantify the overlaps. Scale bar: 100 μm. **b)** We find ~80% overlap between cells that express tdTomato and PDGFRα. Cellomics ArrayScan software (ThermoFisher) was used to quantify the overlaps. Data is presented as mean $\pm$SD. n= 13 images from 3 independent experiments. Source data used to calculate the overlap are provided as a Source Data file. **c)** PD-TT cells at day 77 of differentiation, enriched using A2B5 microbeads were FACS analyzed for the expression of tdTomato. ~27% of A2B5+ cells also express tdTomato. PE in X-axis represents PDGFRα-tdTomato. The same gate (left panel of **c)**) was used for this flow analysis. Gating strategy is further detailed in figure S1j and described in the methods section. **d)** MACS purified and cryopreserved OPCs that were revived and plated on PLO-laminin coated plates for two days. Scale bar: 400 μm. **e)** Cryopreserved cells mature into MBP+ OLs within three weeks in mitogen-free glial media. Scale bar: 200 μm **d)** was independently repeated 5 times and **e)** 2 was independently repeated twice with similar results.

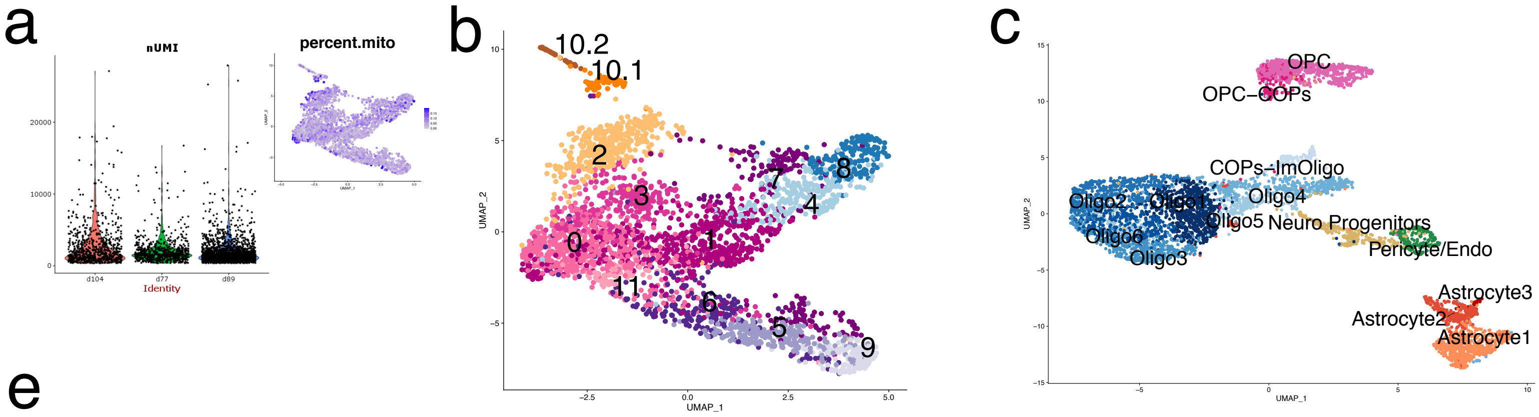

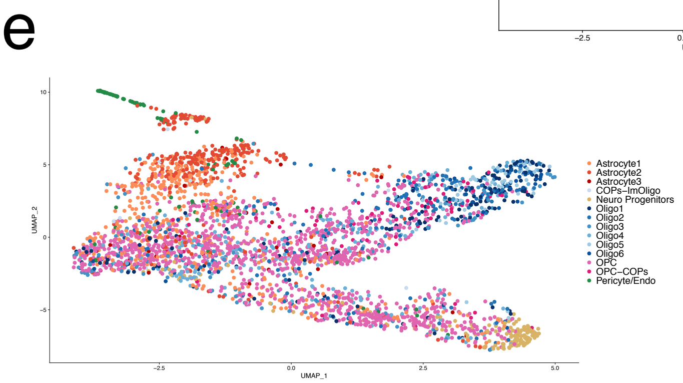

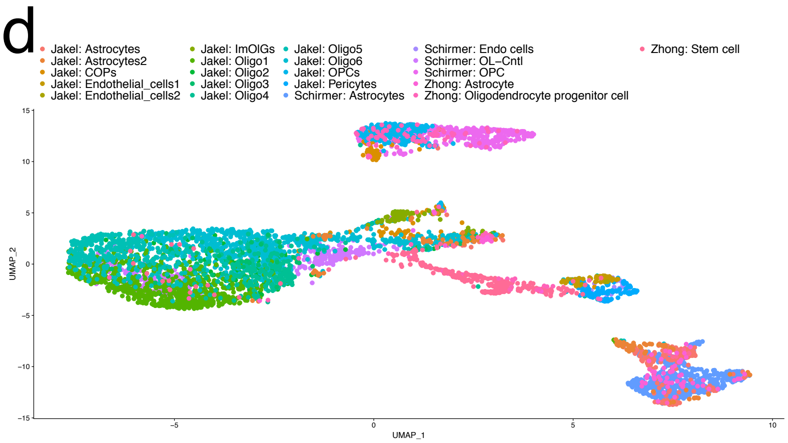

f

Human CNS cells

	endothelial	microglia	neurons	cortex	hippo astros	fetal astros	mature astros	oligos GC	oligos O4
c11	0.015	-0.025	0.388	0.345	0.051	0.412	0.021	0.151	0.357
c10	0.348	0.234	0.062	0.066	0.199	0.413	0.242	0.065	0.111
c9	0.063	0.04	0.282	0.253	0.09	0.458	0.06	0.176	0.361
c8	0.037	0.097	0.263	0.232	0.125	0.32	0.062	0.271	0.424
c7	0.058	0.022	0.285	0.287	0.149	0.419	0.1	0.226	0.42
c6	0.062	0.004	0.285	0.277	0.125	0.445	0.09	0.186	0.385
c5	0.079	0.035	0.28	0.264	0.121	0.47	0.1	0.178	0.368
c4	0.035	0.062	0.284	0.274	0.115	0.346	0.073	0.26	0.431
c3	0.092	0.02	0.264	0.277	0.171	0.425	0.145	0.188	0.388
c2	0.173	0.059	0.209	0.263	0.322	0.485	0.333	0.136	0.306
c1	0.045	0.021	0.322	0.295	0.11	0.403	0.057	0.225	0.422
c0	0.063	0.014	0.307	0.297	0.137	0.417	0.103	0.205	0.406

g

	c0	c1	c2	c3	c4	c5	c6	c7	c8	c9	c10	c11
all oligos	0.428	0.457	0.407	0.42	0.557	0.189	0.268	0.463	0.614	0.163	0.225	0.403
oligos6	0.432	0.459	0.414	0.429	0.565	0.208	0.281	0.47	0.626	0.181	0.242	0.404
oligos5	0.432	0.459	0.414	0.429	0.565	0.208	0.281	0.47	0.626	0.181	0.242	0.404
oligos4	0.418	0.446	0.39	0.407	0.538	0.185	0.263	0.449	0.593	0.16	0.207	0.394
oligos3	0.388	0.416	0.383	0.384	0.518	0.154	0.234	0.431	0.58	0.132	0.198	0.367
oligos2	0.424	0.449	0.409	0.419	0.54	0.193	0.267	0.456	0.592	0.169	0.231	0.393
oligos1	0.393	0.428	0.363	0.382	0.532	0.165	0.24	0.433	0.595	0.142	0.195	0.374
IM oligos	0.398	0.423	0.428	0.399	0.503	0.133	0.22	0.428	0.552	0.115	0.24	0.374
OPCs	0.508	0.543	0.453	0.518	0.568	0.215	0.308	0.52	0.551	0.186	0.18	0.456
all astros	0.371	0.369	0.548	0.392	0.393	0.092	0.175	0.36	0.415	0.055	0.349	0.324
astros2	0.382	0.398	0.476	0.394	0.461	0.102	0.189	0.399	0.505	0.071	0.271	0.345
astros1	0.374	0.362	0.573	0.395	0.36	0.104	0.183	0.342	0.369	0.067	0.383	0.326
peris	0.321	0.312	0.454	0.354	0.301	0.089	0.16	0.27	0.302	0.06	0.531	0.275
endos	0.357	0.348	0.469	0.388	0.337	0.143	0.211	0.312	0.336	0.114	0.497	0.32

value: 0.6, 0.4, 0.2

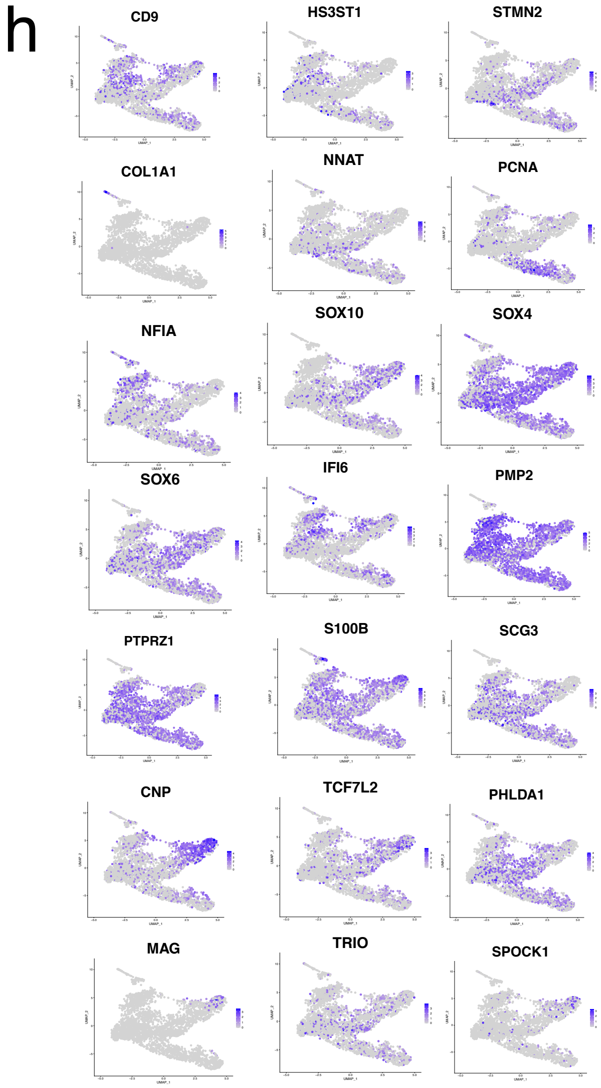

Supplementary Figure 4. Data integration, label prediction and cluster assignment. Quality control metrics applied to the scRNA seq data of MACs purified PD-TT derived OPCs. **a)** Violin plot showing number of UMIs by age and an enrichment heatmap for percent mitochondrial gene content per cell. **b)** Unbiased clustering of the single cell transcriptome divided the cell into 13 clusters (0-9, 10.1, 10.2, and 11). **c-d)** A reference UMAP plot generated by integrating snRNAseq-based transcriptome from three previously published datasets show 15 different clusters **c)** and the source dataset for each cell is labelled **d)**. **e)** Assignment of predicted label to each cell in our dataset. **f and g)** Spearman correlation of single cell transcriptome-based clusters with bulk RNAseq[35] **f)** and snRNAseq[32] **g)** data from primary human brain cells. "All oligos" and "all astros" in **g)** are weighted averages of all 6 oligos or both astro sub-populations. Red color represents higher and blue is used for lower probability. Red color represents higher and blue is used for lower probability. **h)** Distribution of various genetic markers among the different clusters presented as an enrichment heatmap.

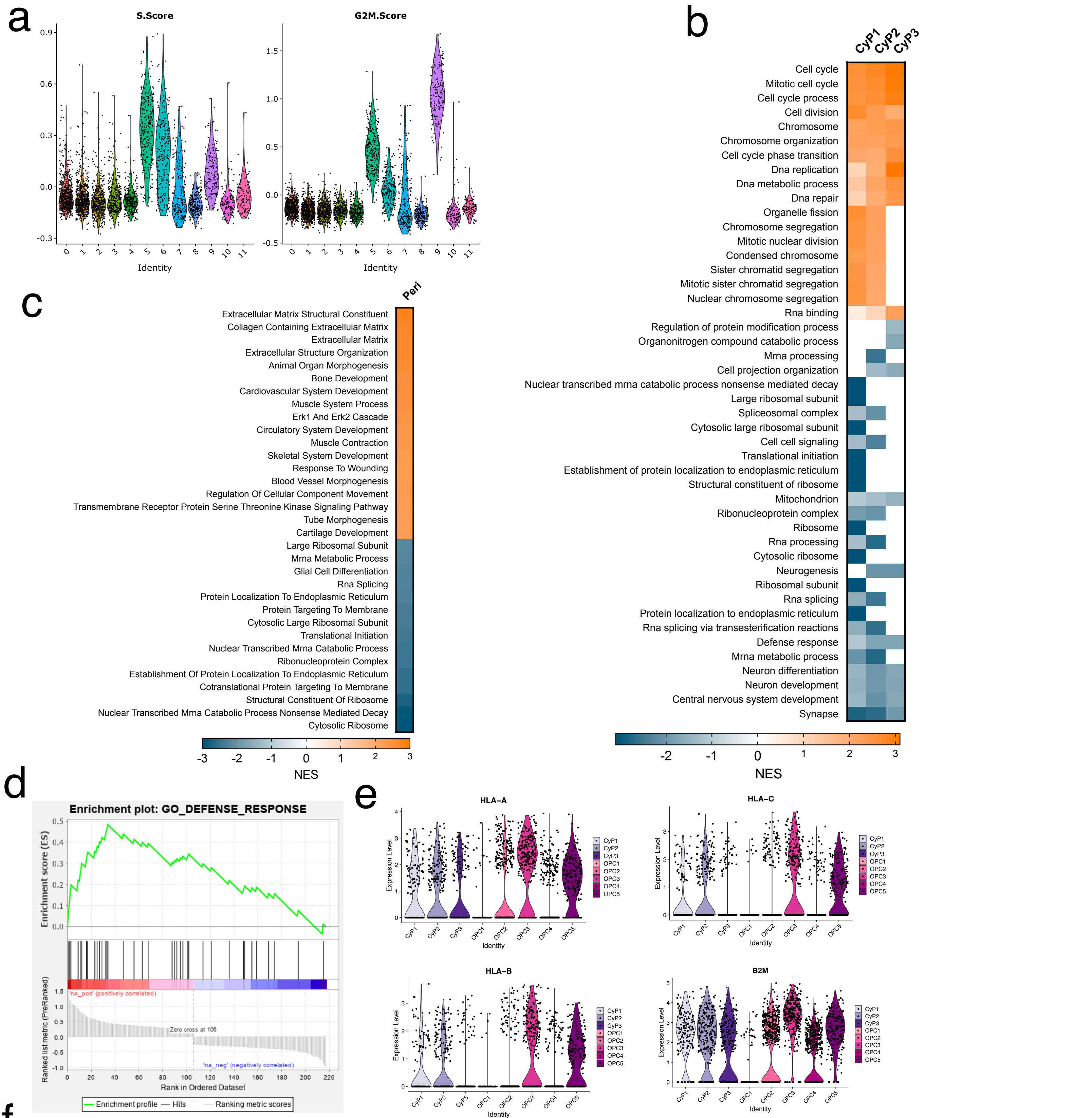

f

Cell Count					
	MBP	PLP 1	GFAP	AQP4	Total cell#
d77	187	248	61	33	691
d89	350	600	553	125	1803
d104	196	321	287	108	777

Percentage				
%	MBP	PLP 1	GFAP	AQP4
d77	27	36	9	5
d89	19	33	31	7
d104	25	41	37	14

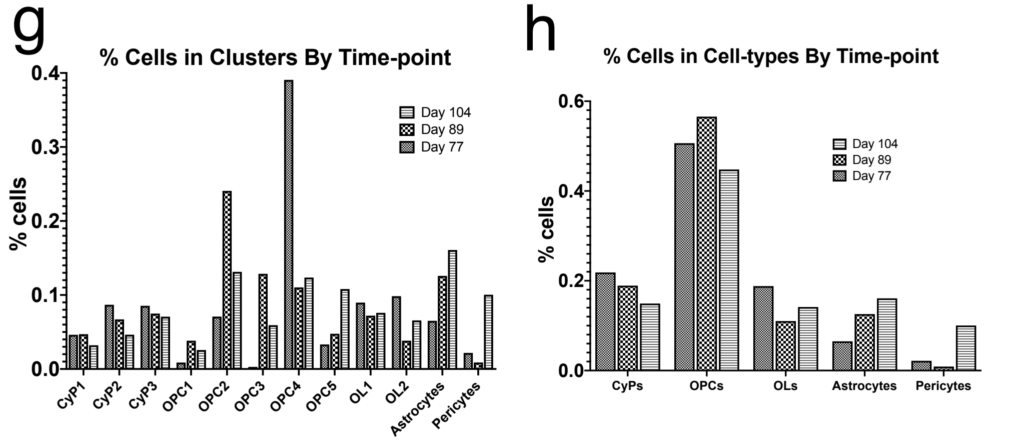

Supplementary Figure 5. GSEA of the scRNAseq data and PDTT reporter cells at different differentiation time-points. a) Violin plot for single cell transcriptome-based cell-cycle analysis showing G2M and S score for each cluster. **b-c)** Heatmap of GSEA highlighting highly enriched pathways among the cycling progenitor **b)** and the pericyte clusters **c)**. Colors indicate significance based on -log10 P-value. Orange color represents upregulated and blue are downregulated pathways. **d)** GSEA enrichment plot for Defense response pathways that is enriched in OPC3. **e)** Violin plot showing the expression of genes responsible for immune and defense responses in different OPC sub-populations. **f)** table showing number and percentage of cells expressing OL and astrocyte markers at different time-points. **g-h)** A bar graph depicting the frequency of each cluster **f)** and cell type **g)** per time point.

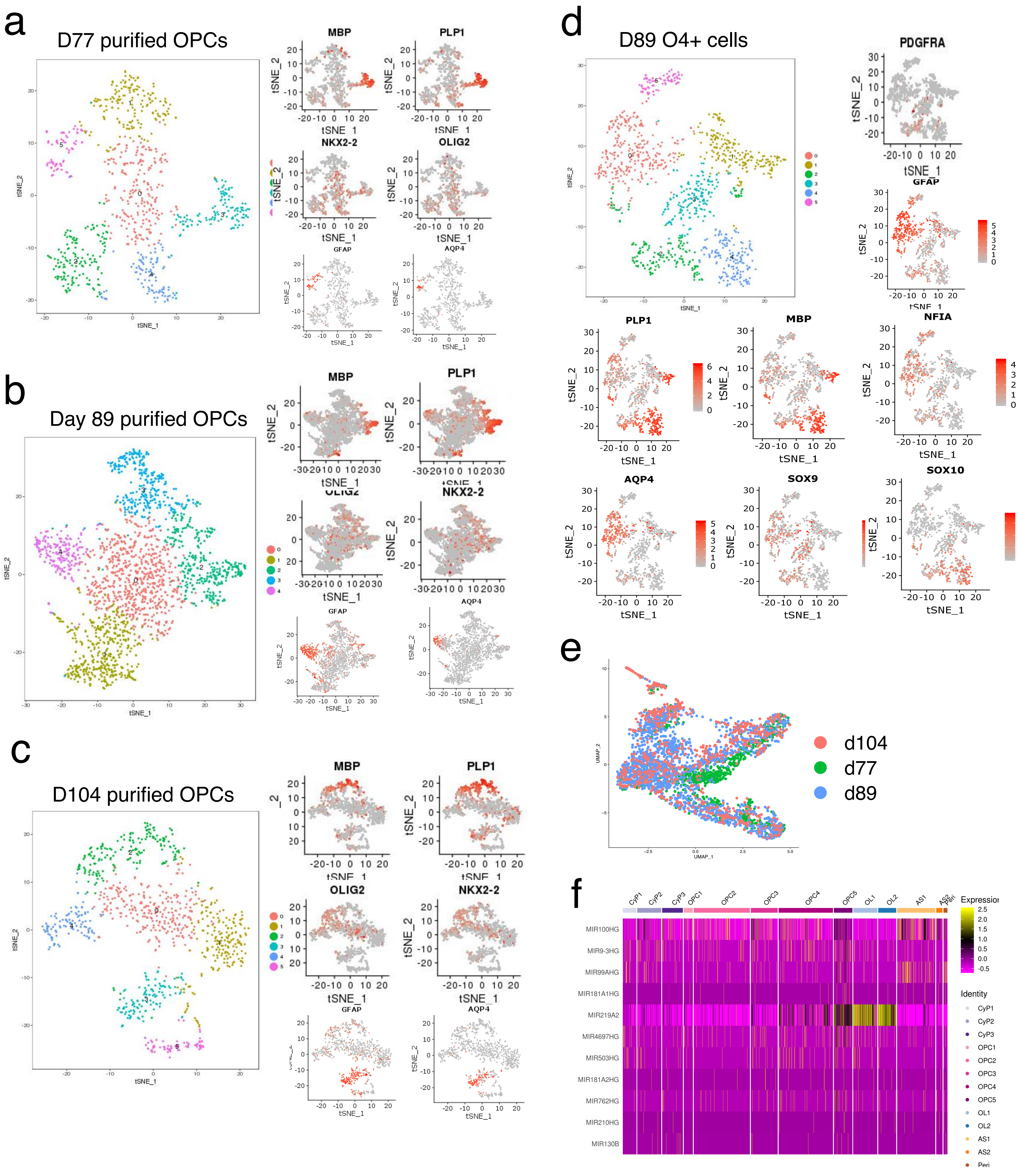

a
D77 purified OPCs
MBP
PLP1
NKX2-2
OLIG2
GFAP
AQP4
b
Day 89 purified OPCs
MBP
PLP1
OLIG2
NKX2-2
GFAP
AQP4
c
D104 purified OPCs
MBP
PLP1
OLIG2
NKX2-2
GFAP
AQP4
d
D89 O4+ cells
PDGFRA
GFAP
PLP1
MBP
NFIA
AQP4
SOX9
SOX10
e
d104
d77
d89
f
CyP1
CyP2
CyP3
OPC1
OPC2
OPC3
OPC4
OPC5
OL1
OL2
AS1
AS2
Peri
MIR100HG
MIR9-3HG
MIR99AHG
MIR181A1HG
MIR219A2
MIR4697HG
MIR503HG
MIR181A2HG
MIR762HG
MIR210HG
MIR130B
Expression
Identity

Supplementary Figure 6. scRNAseq analysis of the purified PDTT reporter OPCs separated by different differentiation time-points. a-c) t-SNE based unsupervised clustering of single cells captured from PDGFRα+/tdTomato+ reporter OPCs at different stages of differentiation: **a)** day 77, **b)** day 89, and **c)** day 104. Cluster specific enrichment of various OL and astrocyte markers indicate OLLC and ALC subgroups of PDGFRα+ cells in each time-point. **d)** PD-TT cells at day 89 of differentiation were MACS purified using O4 beads and processed for scRNAseq with Drop-seq. t-SNE based unsupervised clustering and heatmap showing expression patterns of OL and astrocyte markers of day 89 PDTT cells enriched using O4 microbeads. **e)** Cells in the UMAP plot labelled by the day at which they were purified (day 77, 89, or 104). **f)** Heatmap of pri-microRNAs captured by scRNAseq analysis. miR219 is enriched in OL clusters, miR99AHG and miR100HG are enriched in astrocyte clusters.

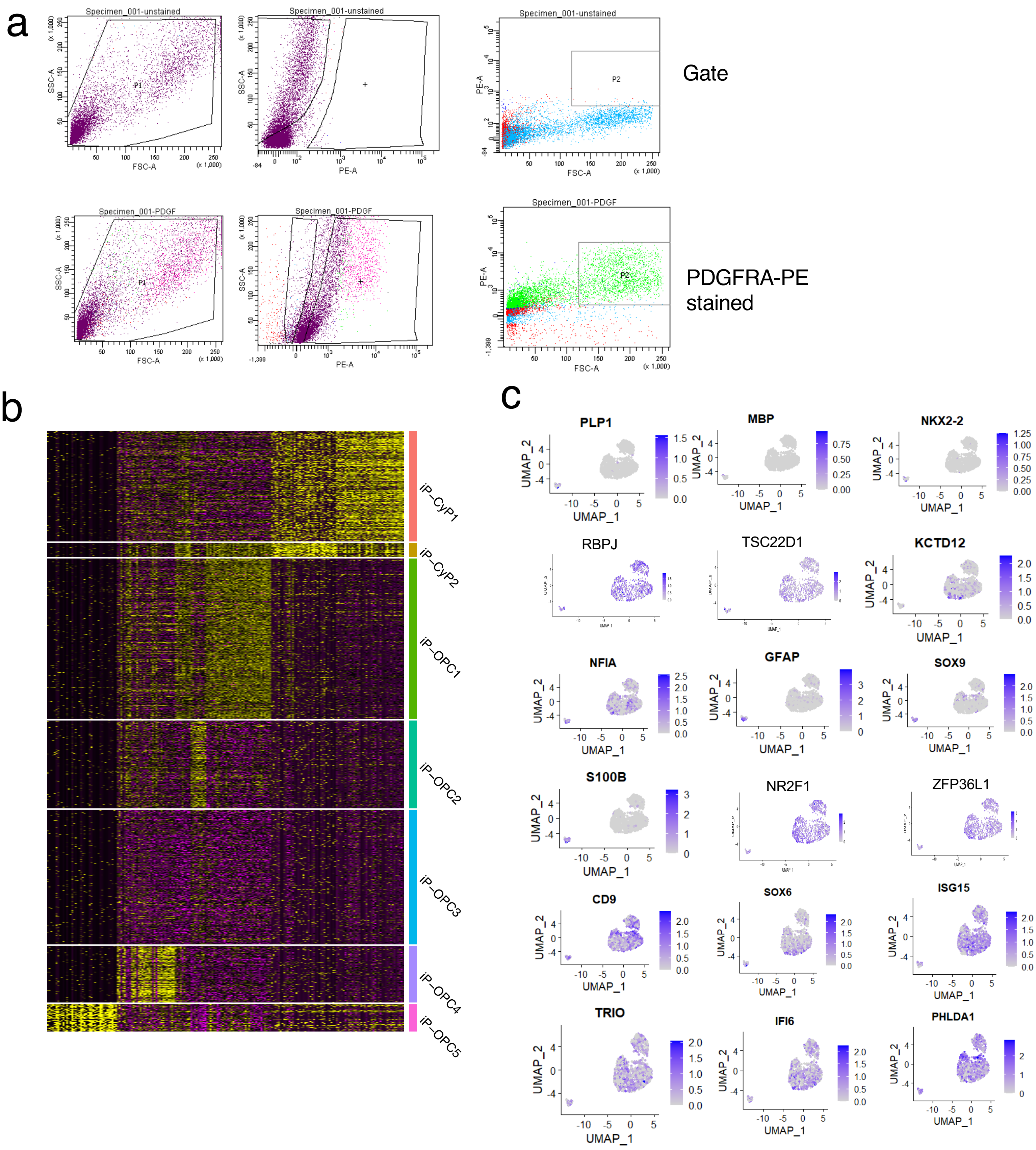

Supplemental Figure 7. Bioinformatic analysis of the hiPSC-derived PDGFRa+ OPCs. a) hiPSC-derived, day 60 OPCs were stained with antibody against PDGFRα and FACS-purified. Top panel shows the gate used for the FACS. Single cells from the FACS-purified samples were captured using the 10X-genomics platform, and their transcriptome was sequenced and analyzed. **b)** An expression heatmap of all the enriched genes from each cluster. **c)** Distribution of various genetic markers among the different clusters presented as an enrichment heatmap.

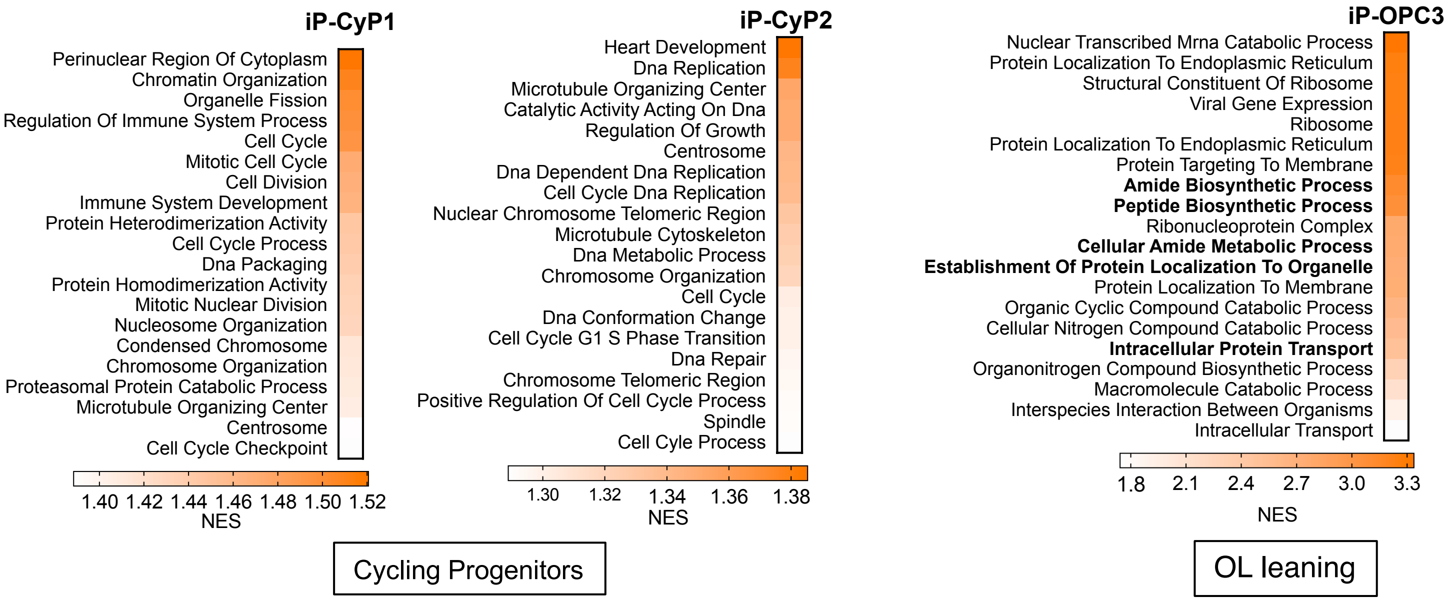

Supplementary Figure 8. Go term analysis of sub-population of hiPSC-derived PDGFRa+ OPCs. Heatmaps of GSEA shows cluster-specific enrichment cell cycle related pathways (iP-Cyp1 and iP-CyP2) and OL-related pathways iP-OPC3. Detailed GSEA for all the clusters are listed in Supplementary Data 7. Orange represents enrichment.

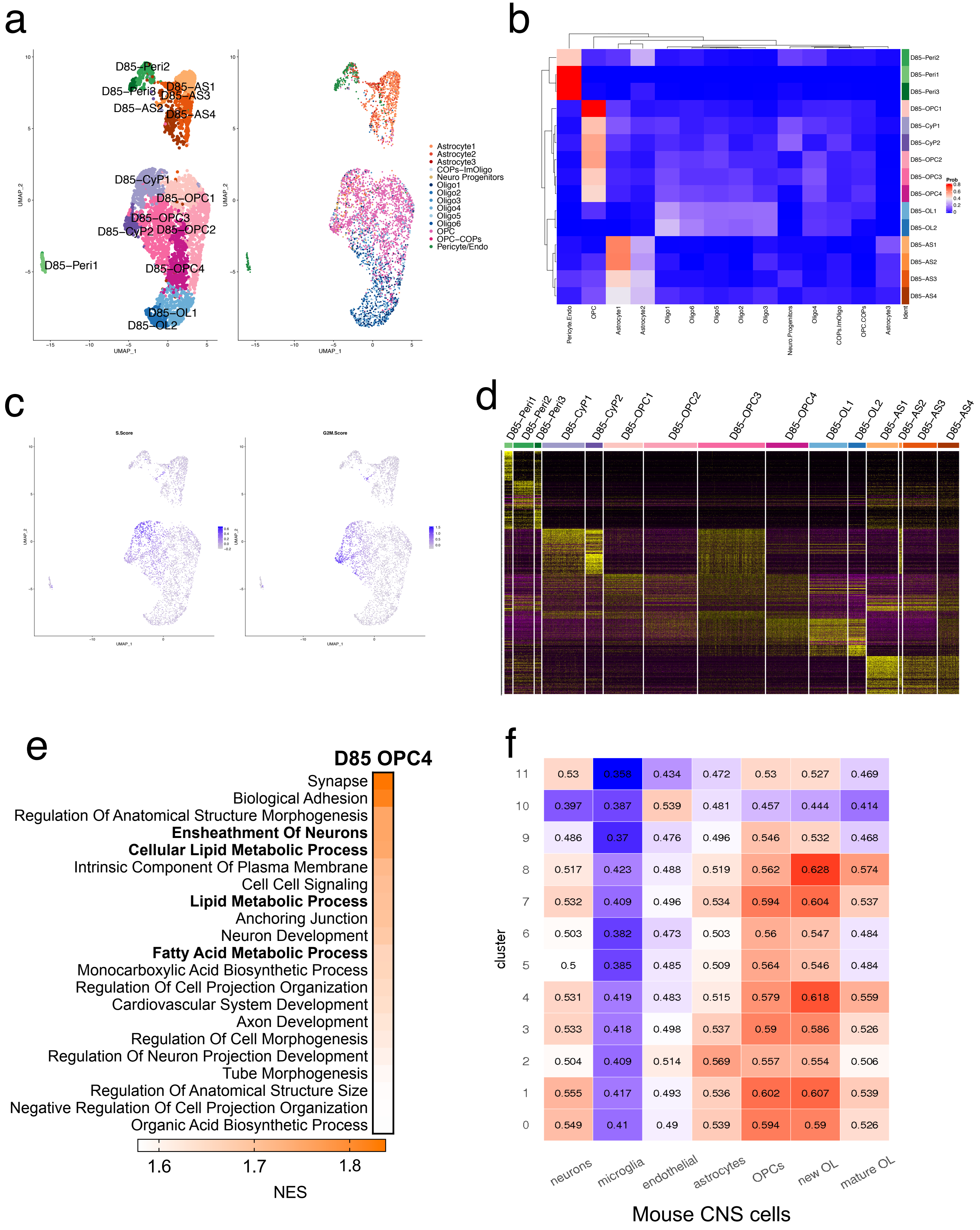
a
D85–Peri2
D85–Peri3
D85–AS1
D85–AS3
D85–AS2
D85–AS4
D85–CyP1
D85–OPC1
D85–OPC3
D85–CyP2
D85–OPC2
D85–Peri1
D85–OPC4
D85–OL1
D85–OL2
UMAP_1
UMAP_2
Astrocyte1
Astrocyte2
Astrocyte3
COPs–ImOligo
Neuro Progenitors
Oligo1
Oligo2
Oligo3
Oligo4
Oligo5
Oligo6
OPC
OPC–COPs
Pericyte/Endo
b
Pericyte.Endo
OPC
Astrocyte1
Astrocyte2
Oligo1
Oligo6
Oligo5
Oligo2
Oligo3
Neuro Progenitors
Oligo4
COPs.ImOligo
OPC.COPs
Astrocyte3
Ident
Prob
c
S.Score
G2M.Score
d
e
D85 OPC4
Synapse
Biological Adhesion
Regulation Of Anatomical Structure Morphogenesis
Ensheathment Of Neurons
Cellular Lipid Metabolic Process
Intrinsic Component Of Plasma Membrane
Cell Cell Signaling
Lipid Metabolic Process
Anchoring Junction
Neuron Development
Fatty Acid Metabolic Process
Monocarboxylic Acid Biosynthetic Process
Regulation Of Cell Projection Organization
Cardiovascular System Development
Axon Development
Regulation Of Cell Morphogenesis
Regulation Of Neuron Projection Development
Tube Morphogenesis
Regulation Of Anatomical Structure Size
Negative Regulation Of Cell Projection Organization
Organic Acid Biosynthetic Process
1.6
1.7
1.8
NES
f
cluster
11 0.53 0.358 0.434 0.472 0.53 0.527 0.469
10 0.397 0.387 0.539 0.481 0.457 0.444 0.414
9 0.486 0.37 0.476 0.496 0.546 0.532 0.468
8 0.517 0.423 0.488 0.519 0.562 0.628 0.574
7 0.532 0.409 0.496 0.534 0.594 0.604 0.537
6 0.503 0.382 0.473 0.503 0.56 0.547 0.484
5 0.5 0.385 0.485 0.509 0.564 0.546 0.484
4 0.531 0.419 0.483 0.515 0.579 0.618 0.559
3 0.533 0.418 0.498 0.537 0.59 0.586 0.526
2 0.504 0.409 0.514 0.569 0.557 0.554 0.506
1 0.555 0.417 0.493 0.536 0.602 0.607 0.539
0 0.549 0.41 0.49 0.539 0.594 0.59 0.526
neurons
microglia
endothelial
astrocytes
OPCs
new OL
mature OL
Mouse CNS cells

Supplementary Figure 9. Single cell transcriptomic analysis of D85 purified OPCs. a) Unsupervised clustering of the single cell transcriptional profiles of D85 purified PDGFRα-tdTomato+ cells from an independent batch of differentiation and assignment of predicted label to each cell in our dataset. **b)** Average cell-type label probability per cluster transferred from the combined reference. Red is used to indicate her and blue is for lower probability. **c)** Cell-cycle analysis showing cells at G2M and S score for each cell. Blue indicates higher score. **d)** An expression heatmap of all the enriched genes from each cluster. Yellow represents enriched expression. **e)** Heatmap of GSEA for OPC sub-clusters highlighting OL-related pathways (OPC4). Detailed GSEA for all the clusters are listed in Supplementary Data 6. Orange represents enrichment. **f)** Spearman correlation of single cell transcriptome-based clusters with RNAseq data from primary mouse brain cells.[3]

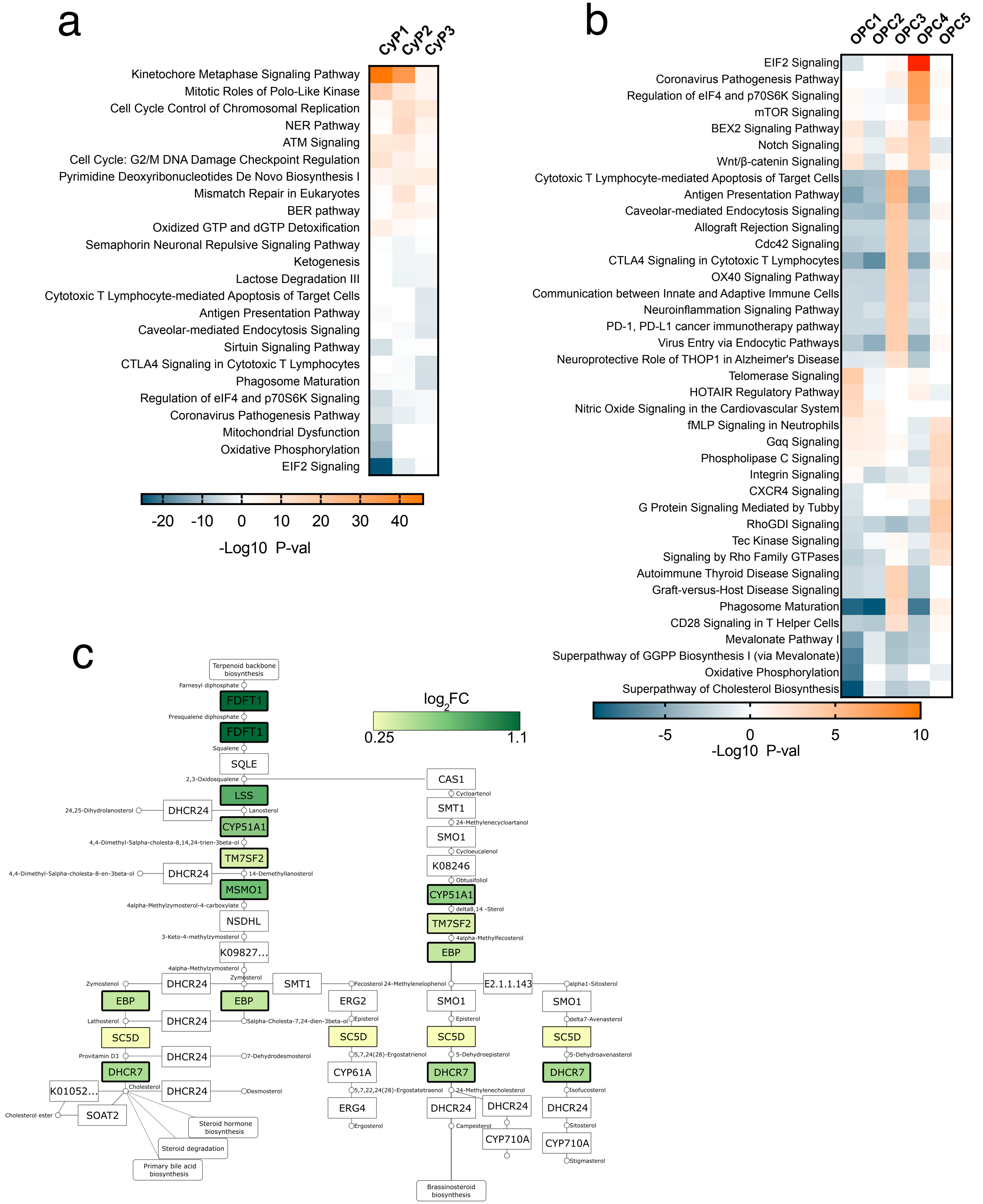

Supplementary Figure 10. **Pathway analysis of the differentially expressed genes**. **a)** Top enriched pathways implicated for **a)** cycling progenitor and **b)** OPC sub-clusters presented as a heatmap. Colors indicate significance based on -log10 P-value. Orange color represents upregulated and blue are downregulated pathways. **c)** A map for KEGG steroid biosynthesis pathway (analogous to cholesterol pathway in IPA), which is highly enriched within the mature OL1 and OL2 clusters, is presented here as an example. Colored boxes denote genes that are significantly enriched based on our RNA-seq data. Green represents higher enrichment.

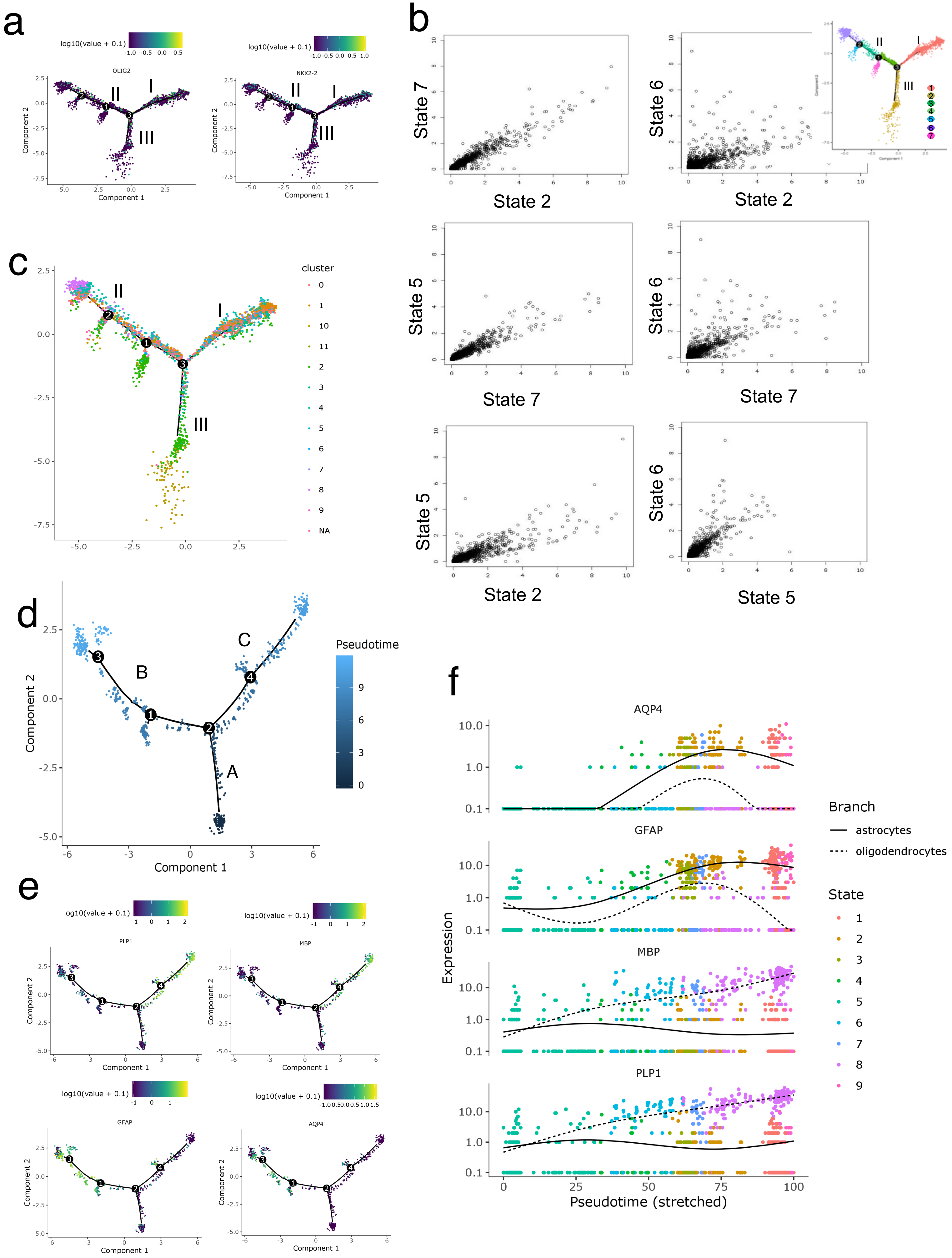
a
log10(value + 0.1)
OLIG2
NKX2-2
Component 1
Component 2
I
II
III
b
State 7
State 2
State 6
State 5
c
cluster
0
1
10
11
2
3
4
5
6
7
8
9
NA
d
Pseudotime
A
B
C
e
PLP1
MBP
GFAP
AQP4
f
AQP4
GFAP
MBP
PLP1
Expression
Pseudotime (stretched)
Branch
astrocytes
oligodendrocytes
State
1
2
3
4
5
6
7
8
9

Supplementary Figure 11. Monocole-2 analysis of the PDTT reporter cells purified with Thy1.2 or O4+ microbeads. a) Within the trajectories derived from the PDTT OPCs purified with Thy1.2 microbeads, OLLC markers (*OLIG2* and *NKX2-2*) show higher expression in Path I and II, but not in Path III or the smaller branches that emerge along Path II. **b)** Scatterplot showing the average gene expression of the cells in astrocyte vs oligodendrocyte states. Each dot represents a gene and axes are average number of transcripts per cell. Cells from the small branches (state 5 and 7) have higher correlation with astrocytes (state 2 cells) than oligodendrocytes (state 6 cells). Inset shows cells at different states. **c)** Overlay of cells from each cluster from figure 4a onto the pseudo-temporal trajectory showing where in the trajectory the cells from each cluster are located. Cells from cluster 0, 1, and 3 make up the majority of Path I population. Cells from astrocyte cluster (cluster 2) make up the majority of population on Path III (state 2 cells) and the sub-branches within Path II (state 5 and 7 cells). Cells from cluster 4 and 8 are located at the end of Path II (state 6 cells). Cells from cluster 10 are located at the end of Path III or state 2 cells. **d)** Developmental trajectories of the O4 microbead enriched day 89 reporter OPCs, generated using Monocole-2. Developmentally younger cells (A) represent OPCs and fall on shorter branch. OPCs have two prominent developmental paths B and C. **e)** Expression of OL and astrocyte markers within the trajectory show enrichment of astrocyte makers (*GFAP, AQP4)* on Path B and OLLC markers (*MBP, PLP1)* on Path C. Green/yellow represents increased expression. **f)** Pseudotemporal expression pattern of OL and astrocyte genes showing kinetic trend of the OL, (*MBP* and *PLP1)* and astrocyte (*GFAP* and *AQP4)* marker genes.

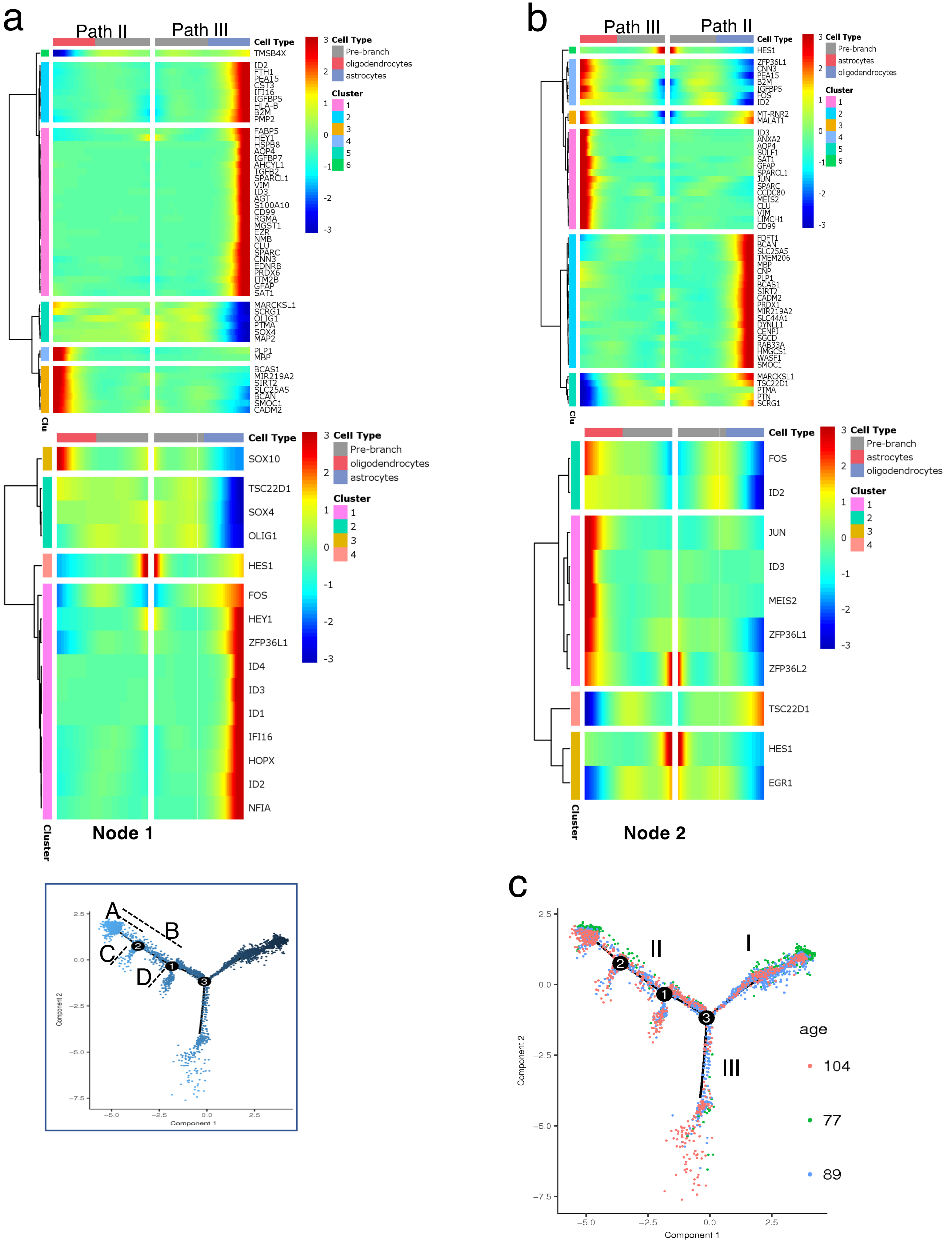
a
Path II
Path III
Cell Type
Pre-branch
oligodendrocytes
astrocytes
Cluster
TMSB4X
ID2
FTH1
PEA15
CST3
IFI16
IGFBP5
HLA-B
B2M
PMP2
FABP5
HEY1
HSPB8
AQP4
IGFBP7
AHCYL1
TGFB2
SPARCL1
VIM
ID3
AGT
S100A10
CD99
RGMA
MGST1
EZR
NMB
CLU
SPARC
CNN3
EDNRB
PRDX6
ITM2B
GFAP
SAT1
MARCKSL1
SCRG1
OLIG1
PTMA
SOX4
MAP2
PLP1
MBP
BCAS1
MIR219A2
SIRT2
SLC25A5
BCAN
SMOC1
CADM2
SOX10
TSC22D1
HES1
FOS
ZFP36L1
ID4
ID1
HOPX
NFIA
Cluster
Node 1
b
JUN
MEIS2
ZFP36L2
EGR1
CNN3
MT-RNR2
MALAT1
ANXA2
SULF1
CCDC80
LIMCH1
FDFT1
TMEM206
CNP
CADM2
PRDX1
SLC44A1
DYNLL1
CENPJ
SGCD
RAB33A
HMGCS1
WASF1
PTN
Node 2
A
B
C
D
Component 1
Component 2
c
I
II
III
age
104
77
89

Supplementary Figure 12. Heatmap of the most differentially expressed genes between different sub-branches of the monocole-2-based trajectories. **a and b)** Differential heatmap capturing the most differentially expressed genes **(a)** and transcription factors **(b)** between Paths II (oligodendrocytes) and III (astrocytes) identified by BEAM and clustered according to their expression pattern. Red color represents upregulation and blue is used for downregulation. Analysis of differential expression at node 2 represents genes from sub-branch A vs C **(b)**, and node 1 represents sub-branch B vs D **(a**). See inset on the bottom left for labelling of nodes and sub-branches. **c)** Cells in each path of the trajectory are labelled by day at which they were purified (day 77, 89, or 104). Although some enrichment of day 104 cells is seen in the mature clusters, each path consists of cells from all three ages.

Supplementary Table 1. **Oligonucleotides used for this study.**

**Primers for Reporter Cell line**	
PDGFRa guide F	caccAGACAGCTTCCTGTAACTGG
PDGFRa guide R	aaacCCAGTTACAGGAAGCTGTCT
PDGFRa homology arm PCR-F	TTGGGGGTCTGCGATGGAAC
PDGFRa homology arm PCR-R	TGGGGTCGCCAAAGCTTTCAT
PDGFRa genotyping-F	GAGACGGGTTCCAGCAGTTCCA
PDGFRa genotyping-R	CATCCTGGCTGCCACACACA
PDGFRa-gibson 1F	TAACTGGCGGATTCGAGGGGTT
PDGFRa-gibson 1R	CAGGAAGCTGTCTTCCACCAGGTCT
PDGFRa-gibson 2F	AGACCTGGTGGAAGACAGCTTCCTGGGGAGCGG AGCTACTAACTTCAGCCT
PDGFRa-gibson 2R	AGGAACCCCTCGAATCCGCCAGTTATCACAGAGA AATGAAGTCCAGGGCTTG
**Off-target Primers**	
Chr16:68022301-F	CCAAGTCAGCTGGGCCTTCTGC
Chr16:68022301-R	GGAGGCTGCGCCTGAAGTCGT
Chr11:113559618-F	GGTCTCCACTCCTCCCCGTACCC
Chr11:113559618-R	GCCCAGGCCAGGGGAGCTAA
Chr10:25104942-F	TGGCCTTGCCACCTGAGAAATGA
Chr10:25104942-R	GCCCAGAAAGGATGGGAGACAGG
Chr2:72465148-F	GCATGCGCCTTTATATACTGAAAATCTTCCTG
Chr2:72465148-R	GACTGTGATATGTATGGCTCAGTATTCCCAGTT
Chr1:65513195-F	TTACAGCATGGTGCCCTCATCCAA
Chr1:65513195-R	TTTTCCAGAGCCCAGGTTCTAATTCTGA
**qTR-PCR primers**	
qhGAPDH_F	GGAGCGAGATCCCTCCAAAAT
qhGAPDH_R	GGCTGTTGTCATACTTCTCATGG
qhPDGFRa_F;	CCTTGGTGGCACCCCTTAC
qhPDGFRa_R	TCCGGTACCCACTCTTGATCTT
qhSOX10_F	CCTCACAGATCGCCTACACC
qhSOX10_R	CATATAGGAGAAGGCCGAGTAGA
qhGFAP_F	CATCGAGATCGCCACCTACA
qhGFAP_R	TCTGCACGGGAATGGTGAT
qhOLIG2_F	GGCGCGCAACTACATCCT
qhOLIG2_R	CGCTCACCAGTCGCTTCAT
qhMBP-F	AAGGCCAGAGACCAGGATTT
qhMBP-R	TCCCTTGAATCCCTTGTGAG
qhENPP6-F	GACCGCCTGAACGTCATTAT
qhENPP6-R	TCACTTGCTGCAGGTCATTC
qhNG2-F	TATGTTGGCCAGACTTGCAT
qhNG2-R	TGCAGGTCTATGTCGGTCAG

Supplementary Table 2. **Key reagents used for the study.**

REAGENT or RESOURCE	SOURCE	IDENTIFIER	Dilution used
**<u>Antibodies</u>**			
Anti-PDGFR alpha	R&D Systems	AF-307-SP	1:200
PE anti-human CD140a (PDGFRalpha)	BioLegend	323505	1:100
Anti-SOX10	R&D Systems	AF2864-SP	1:100
Anti-NKX2.2	DSHB	Cat#74.5A5; RRID:AB_531794	1:50
Anti-MBP	Millipore	Cat#MAB386; RRID:AB_94975	1:100
Anti-OLIG2	Millipore	Cat#AB9610, RRID:AB_570666	1:500
Anti-O4 clone 81	Millipore	MAB345	1:200
Hoechst 33342	ThermoFisher Scientific	H3570	1:10,000
Alexa Fluor 488 goat anti-rabbit IgG	ThermoFisher Scientific	A-11034	1:500
Alexa Fluor 488 goat anti-mouse IgM	ThermoFisher Scientific	A-21042	1:500
Alexa Fluor 647 goat anti-rabbit IgG	ThermoFisher Scientific	A-21245	1:500
Alexa Fluor 647 donkey anti-goat IgG	ThermoFisher Scientific	A-21447	1:500
**<u>Chemicals, Peptides, and Recombinant Proteins</u>**			
mTeSR1	Stem Cell Technologies	5850	
StemFlex	ThermoFisher Scientific	A3349401	
DMEM/F12	ThermoFisher Scientific	11320082	
Accutase	ThermoFisher Scientific	A11105-01	
Antibiotic-Antimycotic	ThermoFisher Scientific	15240062	
Growth Factor Reduced BD MatrigelTM Matrix	BD Biosciences	354230	
Blebbistatin	Sigma	B0560-1MG	
Opti-MEM™ I Reduced Serum Medium	ThermoFisher Scientific	31985088	
CryoStor® CS10	Stem Cell Technologies	7930	
Lipofectamine™ Stem Transfection Reagent	ThermoFisher Scientific	STEM00001	
Puromycin	ThermoFisher Scientific	A11138-03	
N2 supplement	ThermoFisher Scientific	17502-048	
B27 Supplement without vitamin A	ThermoFisher Scientific	12587-010	
SB431542	Stem Cell Technologies	72234	
LDN193189	Stem Cell Technologies	72147	
Smoothened agonist	Sigma	R2625	
All-trans retinoic acid	Millipore	566660	
Recombinant human PDGF-AA	R&D Systems	221-AA-050	

REAGENT or RESOURCE	SOURCE	IDENTIFIER
Recombinant human IGF-I	R&D Systems	291-G1-200
Recombinant human HGF	R&D Systems	294-HG-025
Neurotrophin 3 (NT3)	Millipore	GF031
cAMP analog	Sigma	D0260
3,3,5-Triiodo-l-thyronine (T3)	Sigma	T2877
l-Ascorbic acid	Sigma	A4403
Insulin solution human	Sigma	19278
Biotin	Sigma	4639
Poly-L-ornithine hydrobromide	Sigma	3655
HEPES (1 M)	ThermoFisher Scientific	15630080
Laminin	MilliporeSigma	L2020-1MG
**Critical Commercial Assays**		
MycoAlert Mycoplasma Detection kit	Lonza	LT07-218
hPSC Genetic Analysis Kit	StemCell Technologies	7550
**Deposited Data**		
RNAseq	This paper	https://www.ncbi.nlm.nih.gov/geo/query/acc.cgi?acc=GSE146373)
Human reference genome NCBI build 37, GRCh37	Genome Reference Consortium	http://www.ncbi.nlm.nih.gov/projects/genome/assembly/grc/human/
RNAseq on the human and mouse CNS cells	Barres Lab	GSE52564, and GSE73721
Human single cell data		GEO database (GSE118257, GSE104276), Bioproject (544731)
**Experimental Models: Cell Lines**		
Passage 40 hESC (WA09) or H9 NIH HES # 0062	WiCell	RRID:CVCL_9773
hiPCS	Dr. Katie Whartenby Lab	
PD-TT (the PDGFRa-tdTomato-Thy1.2 reporter)	This paper	
**Recombinant DNA**		
PX459.V2	Addgene	RRID:Addgene_62988
ZeroBlunt TOPO	ThermoFisher Scientific	K280020
