# Supplementary material for: Single-cell transcriptomic reveals molecular diversity and developmental heterogeneity of human stem cell-derived oligodendrocyte lineage cells
Source: Nat Commun. 2021 Jan 28;12:652. doi: 10.1038/s41467-021-20892-3 (PMC7844020; doi:10.1038/s41467-021-20892-3)
Supplement: Supplementary file 2 — Reporting Summary [file 41467_2021_20892_MOESM2_ESM.pdf]

nature research

Corresponding author(s): Don Zack

Last updated by author(s): Dec 22, 2020

Reporting Summary

Nature Research wishes to improve the reproducibility of the work that we publish. This form provides structure for consistency and transparency in reporting. For further information on Nature Research policies, see Authors & Referees and the Editorial Policy Checklist .

Statistics

For all statistical analyses, confirm that the following items are present in the figure legend, table legend, main text, or Methods section.

n/a	Confirmed	
☐	☒	The exact sample size (*n*) for each experimental group/condition, given as a discrete number and unit of measurement
☐	☒	A statement on whether measurements were taken from distinct samples or whether the same sample was measured repeatedly
☐	☒	The statistical test(s) used AND whether they are one- or two-sided<br>*Only common tests should be described solely by name; describe more complex techniques in the Methods section.*
☒	☐	A description of all covariates tested
☐	☒	A description of any assumptions or corrections, such as tests of normality and adjustment for multiple comparisons
☐	☒	A full description of the statistical parameters including central tendency (e.g. means) or other basic estimates (e.g. regression coefficient) AND variation (e.g. standard deviation) or associated estimates of uncertainty (e.g. confidence intervals)
☐	☒	For null hypothesis testing, the test statistic (e.g. *F*, *t*, *r*) with confidence intervals, effect sizes, degrees of freedom and *P* value noted<br>*Give P values as exact values whenever suitable.*
☒	☐	For Bayesian analysis, information on the choice of priors and Markov chain Monte Carlo settings
☒	☐	For hierarchical and complex designs, identification of the appropriate level for tests and full reporting of outcomes
☒	☐	Estimates of effect sizes (e.g. Cohen's *d*, Pearson's *r*), indicating how they were calculated

Our web collection on statistics for biologists contains articles on many of the points above.

Software and code

Policy information about availability of computer code

Data collection	No software was used
Data analysis	To extract expression matrices from fastq files dropseq cookbook (version 1.0.1) was used. The principal component analysis (PCA), t-distributed stochastic neighbor embedding (tSNE) and UMAP analyses were performed using a previously published, open source, R package, Seurat (version 2 and 3). Time-series analysis to generate a pseudotemporal trajectory was performed using an unsupervised differential gene expression test based on sample age in Monocle (version 2). The networks and pathway analyses were generated through the use of Gene Set Enrichment Analysis, Ingenuity Pathway Analysis (IPA QIAGEN Inc) and KEGG via Visualization and Integrated Discovery (DAVID, v6.7) tools. qPCR analysis was performed using CFX Maestro version: 4.1.2434.0124 (Biorad). Graphs were generated using prism version 9. Co-staining analysis of the tdTomato and PDGFRA was performed using a built-in algorithm of the ArrayScan image analysis software (ThermoFisher Scientific ArrayScan XTI). For flow analysis SH-800 Cell Sorter and its built-in software, SH800S was used.<br><br>NO custom codes were developed in this study. The previously published codes used in this study are deposited to: https://github.com/wefang/TdTom

For manuscripts utilizing custom algorithms or software that are central to the research but not yet described in published literature, software must be made available to editors/reviewers. We strongly encourage code deposition in a community repository (e.g. GitHub). See the Nature Research guidelines for submitting code & software for further information.

Data

Policy information about availability of data

All manuscripts must include a data availability statement. This statement should provide the following information, where applicable:

- Accession codes, unique identifiers, or web links for publicly available datasets
- A list of figures that have associated raw data
- A description of any restrictions on data availability

RNA-Seq data generated for this paper is deposited to the NCBI's Gene Expression Omnibus (GEO) database (GSE146373 (https://www.ncbi.nlm.nih.gov/geo/query/acc.cgi?acc=GSE146373)) and is available for viewing at http://zacklab.org/OPCs/. Previously published, publicly available datasets: GEO database (GSE118257, GSE104276, GSE52564, and GSE73721) and Bioproject (544731) were also used for this study.

Field-specific reporting

Please select the one below that is the best fit for your research. If you are not sure, read the appropriate sections before making your selection.

☒ Life sciences ☐ Behavioural & social sciences ☐ Ecological, evolutionary & environmental sciences

For a reference copy of the document with all sections, see nature.com/documents/nr-reporting-summary-flat.pdf

Life sciences study design

All studies must disclose on these points even when the disclosure is negative.

Sample size	Three samples (three time-points, roughly two weeks apart i.e day 77, 89, and 104 of differentiation) was used for the primary dropseq experiment. The human stem cell-derived OPCs populate a differentiation culture around day 75 and a number of those OPCs mature into oligodendrotyes by day 100. Therefore, the three time-points chosen would sufficiently capture maturation of the PDGFRA-expressing OPCs. More details regarding the choice of these exact time-points is included in the method section, under "Drop-seq-based single cell capture and RNA-sequencing".
Data exclusions	single cells with <250 mRNA molecules and >20% mitochondrial RNA were removed as quality control. This was performed for all scRNAseq data.
Replication	scRNAseq was validated using independent batch of reporter OPCs differentiated to D85 and day 60 hiPSC-derived OPCs. For flow, qPCR, image analysis and drug treatment, cells differentiated as different batches were used as biological replicates. At least two technical replicates were run for each sample in the qPCR and immunohistochemistry. All attempts at replication were successful.
Randomization	not relevant because there were no experimental groups
Blinding	not relevant because there were no experimental groups

Reporting for specific materials, systems and methods

We require information from authors about some types of materials, experimental systems and methods used in many studies. Here, indicate whether each material, system or method listed is relevant to your study. If you are not sure if a list item applies to your research, read the appropriate section before selecting a response.

Materials & experimental systems

n/a	Involved in the study	
☐	☒	Antibodies
☐	☒	Eukaryotic cell lines
☒	☐	Palaeontology
☒	☐	Animals and other organisms
☒	☐	Human research participants
☒	☐	Clinical data

Methods

n/a	Involved in the study	
☒	☐	ChIP-seq
☐	☒	Flow cytometry
☒	☐	MRI-based neuroimaging

Antibodies

Antibodies used	Anti-PDGFR alpha (R&D Systems AF-307-SP)- 1:200;<br>PE anti-human CD140a (BioLegend, 323505)- 1:100;<br>Anti-SOX10 (R&D Systems, AF2864-SP) - 1:100<br>Anti-NKX2.2 (DSHB, 74.5A5); RRID:AB_531794)- 1:50;<br>Anti-MBP Millipore (Cat# MAB386; RRID:AB_94975)- 1:100

Anti-OLIG2 (Millipore Cat# AB9610, RRID:AB_570666)- 1:500;
Anti-O4 (clone 81 Millipore MAB345)- 1-200;
Hoechst 33342 ThermoFisher Scientific H3570- 1:10,000

(All secondary antibodies below were used at 1:500)
Alexa Fluor 488 goat anti-rabbit IgG ThermoFisher Scientific A-11034
Alexa Fluor 488 goat anti-mouse IgM ThermoFisher Scientific A-21042
Alexa Fluor 647 goat anti-rabbit IgG ThermoFisher Scientific A-21245
Alexa Fluor 647 donkey anti-goat IgG ThermoFisher Scientific A-21447

**Antibody list is also available supplementary table 2.

Validation	Anti-PDGFR alpha (R&D Systems AF-307-SP): validated in recombinant human (rh) PDGF R beta (website)<br>PE anti-human CD140a (BioLegend, 323505): validated NIH 3T3 cells transfected with human PDGFRalpha (website)<br>Anti-SOX10 (R&D Systems, AF2864-SP), Anti-NKX2.2 (DSHB, 74.5A5), Anti-MBP Millipore (Cat# MAB386; RRID:AB_94975), Anti-OLIG2 (Millipore Cat# AB9610, RRID:AB_570666), and Anti-O4 (clone 81 Millipore MAB345) were validated in the human plupotent stem cell derived OPCs and oligodendrocytes in the previous publication (Douvaras P. et al. Stem cell reports 2014 and Douvaras, P. &Fossati, V. Nat protoc 2015).

Eukaryotic cell lines

Policy information about cell lines

Cell line source(s)	Passage 40 hESC (WA09, WiCell)- NIH HES # 0062,<br>hiPCS -Dr. Katie Whartenby Lab at Johns Hopkins University School of Medicine.
Authentication	WiCell, Karyotype analysis was performed using a qPCR based hPSC Genetic Analysis Kit (StemCell Technologies, #07550) or by G-banding (CellLine Genetics).
Mycoplasma contamination	Cells were routinely tested for mycoplasma contamination (MycoAlert, Lonza) and the cells were free of contamination.
Commonly misidentified lines (See ICLAC register)	No commonly misidentified cell lines were used in the study.

Flow Cytometry

Plots

Confirm that:

- [x] The axis labels state the marker and fluorochrome used (e.g. CD4-FITC).
- [x] The axis scales are clearly visible. Include numbers along axes only for bottom left plot of group (a 'group' is an analysis of identical markers).
- [x] All plots are contour plots with outliers or pseudocolor plots.
- [x] A numerical value for number of cells or percentage (with statistics) is provided.

Methodology

Sample preparation	hESC-derive OPCs were dissociated into single cell suspension by incubating in accutase for ~45 minutes. The single cell suspension was then passed through a 70 uM cell strainer (BD Biosciences), washed, and resuspended in Live Cell Imaging Solution (ThermoFisher Scientific).
Instrument	SH-800 Cell Sorter (Sony Biotechnology, San Jose, CA)
Software	SH-800S software
Cell population abundance	>10,000 cells per fraction. Purity of sample was accessed by observing and quantifying the cells under fluorescent microscope.
Gating strategy	A gate was setup using WT hES cells differentiated to day 95. Since it is not practical to differentiate a WT line every time a reporter line is differentiated, the same gate was saved and used for every flow analysis. For each analysis, a combination of BSC-A with FSC-A followed by FSC-H with FSC-A were used to subset live cells (Supplemental Fig. 1j and Supplemental Fig. 7a).

- [x] Tick this box to confirm that a figure exemplifying the gating strategy is provided in the Supplementary Information.
