# Supplementary material for: Single-cell transcriptomic reveals molecular diversity and developmental heterogeneity of human stem cell-derived oligodendrocyte lineage cells
Source: Nat Commun. 2021 Jan 28;12:652. doi: 10.1038/s41467-021-20892-3 (PMC7844020; doi:10.1038/s41467-021-20892-3)
Supplement: Supplementary file 3 — Description of Additional Supplementary Files [file 41467_2021_20892_MOESM3_ESM.pdf]

Description of Additional Supplementary Files

Supplementary Movie 1. A 6 hour long time-lapse video of the PD-TT reporter cells at day 45 of differentiation. First few tdTomato+ OPCs are seen migrating out of the neurosphere (bottom of the image) plated on a surface coated with poly-L-ornithine and laminin.

Supplementary Movie 2. Time-lapse video of the PD-TT reporter cells at day 65 of differentiation taken over 18 hours. Numerous tdTomato+ OPCs have migrated out of the neurosphere (at the center of the image) and are seen moving around on a coverslip coated with poly-L-ornithine and laminin.

Supplementary Data 1. List of the differentially expressed genes from each cluster.

Supplementary Data 2. List of the differentially expressed genes between the individual OPC sub-clusters (i.e., CyP2, CyP2, and OPC1-5).

Supplementary Data 3. List of the all the pathways produced by Gene Set Enrichment Analysis for each cluster. For the analysis restricted only to OPC sub-clusters, both upregulated and downregulated pathways are included on the same sheet.

Supplementary Data 4. List of the positively and negatively regulated canonical pathways and their associated transcripts for each cluster.

Supplementary Data 5. Quality control dataset listing number of genes, UMIs and %mitochondrial reads for each cell. A table with number of cells for each cluster per time-point is also included.

Supplementary Data 6. List of all the differentially expressed genes and all the pathways produced by Gene Set Enrichment Analysis for each cluster of D85 PDTT OLLCs.

Supplementary Data 7. List of all the differentially expressed genes and all the pathways produced by Gene Set Enrichment Analysis for each cluster of iPSC-OPCs.
